# Styrylpyridinium Derivatives for Fluorescent Cell Imaging

**DOI:** 10.3390/ph16091245

**Published:** 2023-09-04

**Authors:** Reinis Putralis, Ksenija Korotkaja, Martins Kaukulis, Zhanna Rudevica, Juris Jansons, Olga Nilova, Martins Rucins, Laura Krasnova, Ilona Domracheva, Mara Plotniece, Karlis Pajuste, Arkadij Sobolev, Felikss Rumnieks, Laura Bekere, Anna Zajakina, Aiva Plotniece, Gunars Duburs

**Affiliations:** 1 Latvian Institute of Organic Synthesis, LV-1006 Riga, Latvia; reinis.putralis@osi.lv (R.P.); martins.kaukulis@osi.lv (M.K.); rucins@osi.lv (M.R.); chernova@osi.lv (L.K.); ilona@farm.osi.lv (I.D.); kpajuste@osi.lv (K.P.); arkady@osi.lv (A.S.); laura.bekere.8@gmail.com (L.B.); 2 Department of Pharmaceutical Chemistry, Faculty of Pharmacy, Riga Stradiņš University, LV-1007 Riga, Latvia; mara.plotniece@rsu.lv; 3 Latvian Biomedical Research and Study Centre, LV-1067 Riga, Latvia; ksenija.korotkaja@biomed.lu.lv (K.K.); zanna.rudevica@biomed.lu.lv (Z.R.); jansons@biomed.lu.lv (J.J.); nilovaa.olga@gmail.com (O.N.); felikss.rumnieks@biomed.lu.lv (F.R.); anna@biomed.lu.lv (A.Z.); 4 Faculty of Materials Science and Applied Chemistry, Riga Technical University, LV-1048 Riga, Latvia

**Keywords:** styrylpyridines, julolidine, self-assembly, near-infrared fluorescent dye, cell labelling, macrophages

## Abstract

A set of styrylpyridinium (SP) compounds was synthesised in order to study their spectroscopic and cell labelling properties. The compounds comprised different electron donating parts (julolidine, *p*-dimethylaminophenyl, *p*-methoxyphenyl, 3,4,5-trimethoxyphenyl), conjugated linkers (vinyl, divinyl), and an electron-withdrawing *N*-alkylpyridinium part. Geminal or *bis*-compounds incorporating two styrylpyridinium (*bis*-SP) moieties at the 1,3-trimethylene unit were synthesised. Compounds comprising a divinyl linker and powerful electron-donating julolidine donor parts possessed intensive fluorescence in the near-infrared region (maximum at ~760 nm). The compounds had rather high cytotoxicity towards the cancerous cell lines HT-1080 and MH-22A; at the same time, basal cytotoxicity towards the NIH3T3 fibroblast cell line ranged from toxic to harmful. SP compound **6e** had IC_50_ values of 1.0 ± 0.03 µg/mL to the cell line HT-1080 and 0.4 µg/mL to MH-22A; however, the basal toxicity LD_50_ was 477 mg/kg (harmful). The compounds showed large Stokes’ shifts, including 195 nm for **6a**,**b**, 240 nm for **6e**, and 325 and 352 nm for **6d** and **6c**, respectively. The highest photoluminescence quantum yield (PLQY) values were observed for **6a**,**b**, which were 15.1 and 12.2%, respectively. The PLQY values for the SP derivatives **6d**,**e** (those with a julolidinyl moiety) were 0.5 and 0.7%, respectively. Cell staining with compound **6e** revealed a strong fluorescent signal localised in the cell cytoplasm, whereas the cell nuclei were not stained. SP compound **6e** possessed self-assembling properties and formed liposomes with an average diameter of 118 nm. The obtained novel data on near-infrared fluorescent probes could be useful for the development of biocompatible dyes for biomedical applications.

## 1. Introduction

The styryl fragment (Ph-CH=CH-) is a common moiety included in the structures of different pharmaceuticals, polymers, agrochemicals, fluorophores, dyes, and materials. It is one of the most common functional groups in biologically active organic compound structures [1,2]. Styryl dyes have high-tech applications as materials for light emission and photoelectric and photochemical activity [1,3]. Among various styryl compounds, two important classes of natural products are mentioned—stilbenes, including resveratrol derivatives [4], and chalcones, including curcumin derivatives [5]—as the main representatives. 4-*N*,*N*-Dimethylaminostyryl-1-methylpyridinium tosylate [6] is converted to an important optoelectronic material [7]. Styrylpyridinium tosylates (and also bromides or chlorides) are widely proposed as fluorescent probes for biochemical, biophysical, and molecular biology studies [6,8,9,10,11]. Large Stokes’ shift styryl dyes were mentioned as bio-imaging agents and have been widely studied during the last decade. For example, cyanine–styryl derivatives with Stokes’ shifts larger than 70 nm were proposed as DNA binding agents and also displayed anti-bacterial and anti-cancer properties [12]. A highly fluorescent styrylpyridinium–pyrene hybrid with a large Stokes’ shift of around 130 nm showed remarkable selectivity to stain the nucleus in both live and fixed cells [13]. Other styrylpyridinium dyes with Stokes’ shifts of more than 200 nm and improved fluorescence quantum yield have been proposed as useful reagents for the colorimetric detection of fluoride ions [14].

It was previously demonstrated that the fluorescence of a group of styrylpyridinium (SP) probes increased after an association with glucose oxidase (GOx), resulting in blue shifts up to 30 nm of the fluorescence emission maxima [15]. Pronounced changes in the fluorescence intensities of styrylpyridinium derivatives in the presence of GOx suggested that the enzyme readily binds to the probes via non-covalent interactions. Cationic styryl dyes show a wide range of F_max_ values with a change in the electron-withdrawing (EWG) properties of a cationic heteroaromatic ring and electron-donating force of another aromatic ring, making them good candidates for application as solid-state fluorescent materials [16]. It was confirmed that the strong electron acceptor–donor system that was provided by the electron-donating *N*,*N*-dialkyl group in the molecule increased the electron flow towards the electron acceptor moieties. The long π conjugation style, length of the conjugated bridge, and molecular planarity are essential preconditions for the design of functional chromophores [17,18,19]. The synthesis and evaluation of the physicochemical and self-assembling properties of styrylpyridinium moieties containing a 1,4-DHP amphiphile as a prospective delivery system were demonstrated by our research group [20]. It was demonstrated that the structure of SP or related derivatives affected their properties. Thus, a change in the nature of the donor and acceptor units and that of the π-linker led to an intense increase in hyperpolarisability after grooving of the molecular conjugation, whereas the hyperpolarisability was almost unaffected by an increase in the donor/acceptor strength [18].

Julolidine-2,3,6,7-tetrahydro-1H,5Hbenzo[1,2]quinolizine and its derivatives are a structural subunit widely used in synthesis of fluorescent dyes [21]. The structure of julolidine is formally proposed as dipropylaniline with two fixed moieties and is defined as an electron-donating group in fluorescent dyes. Julolidine derivatives have shown potential applications in various fields of science and technology due to their fluorescence properties [22,23].

In recent decades, styrylpyridinium has also been shown to have biological properties, including antimicrobial properties against bacteria [24,25,26] and antifungal activity for effective inhibition of cells of the pathogenic yeast *Candida albicans* [8,9]. The styrylpyridinium derivative 4-(4-cyanostyryl)-1-dodecylpyridin-1-ium (CSDP+) bromide has shown low toxicity on mammalian Hek-293 cells and reduces *Candida albicans* adhesion [27]. Additionally, various styryl dyes have been proposed as effective probes for DNA, RNA, β-amyloid peptides, chymotrypsin, heparin, and other bioanalytes [28,29]. *Bis*-piperazinyl-styrylpyridium-bromobutenyl salt (Br-PSP-PSP-Br) was synthesised and proposed as a sensitive and rapid diagnostic system for avian influenza virus recognition [30]. The cytotoxic assay of hydroxyl and nitro styryl-quinoline derivatives demonstrated that the hydroxy analogues exhibited better cytotoxicity than the nitro analogues [31]. It was concluded that the presence of an EWG at the styryl ring of the molecule is necessary for increasing the cytotoxicity levels [31]. Furthermore, julolidine derivatives were shown to inhibit amyloid B protein self-assembly, which is regarded as a prime strategy for Alzheimer’s disease treatment [32].

The fluorescent properties of the julolidine-based compounds were studied to visualise the accumulation and distribution of these compounds within the cell, aiming to understand their potential anti-parasitic effect. Julolidine-based fluorescent compounds possessed trypanosome alternative oxidase (TAO) inhibition activity in the submicromolar range and accumulated specifically in the mitochondria of mammalian and trypanosome cells [33]. The fluorescent labelling of cells or drug formulations represents an advanced approach for monitoring the treatment results in many diseases, including cancer [34]. Cell tracking and cell migration analyses are important for cancer monitoring and tracking immune cell migration [35,36]. These analyses can provide valuable insights into the dynamic changes in the tumour microenvironment, metastasis, and the interaction between cancer cells and immune cells [37]. By understanding cell migration patterns, novel strategies for cancer treatment, immunotherapy, and targeted drug delivery can be developed.

In this work, we designed new styrylpyridinium (SP) compounds possessing electron-donating substituents at the phenyl moiety and variation in the length of the alkyl chain at the EWG *N*-alkylpyridinium fragment by introducing conjugated vinyl or divinyl linkers. Additionally, geminal or *bis*-styrylpyridinium (*bis*-SP) derivatives with two SP moieties conjugated through the 1,3-trimethylene unit were obtained, combining two geminal fluorescent moieties in the same molecule. In total, nine compounds were synthesised and their spectral, self-assembling, and biological properties were assessed. For evaluation of the biological properties, cytotoxicity assessment was performed. In addition, the cell labelling properties of one selected compound (**6e**) were evaluated using fluorescence microscopy and flow cytometry analysis.

## 2. Results and Discussion

### 2.1. Chemistry

#### 2.1.1. Design

**Figure 1:**
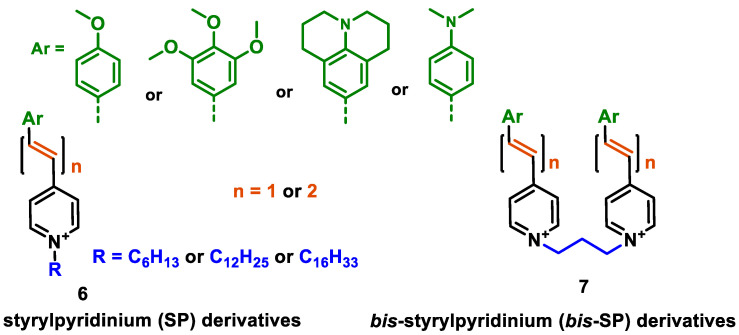
Designed structures of studied styrylpyridinium derivatives **6** and **7**.

The design of the SP derivatives for this study was performed by changing the main structural parameters in the following ways (Figure 1):

1. Modification of the electron-donating part (*p*-methoxyphenyl, 3,4,5-trimethoxyphenyl, *p*-dimethylaminophenyl or julolidine moieties);
2. Modification of the conjugated linker (vinyl, divinyl);
3. Modification of the electron-withdrawing *N*-alkylpyridinium part, introducing dodecyl or hexadecyl chains;
4. Geminal or *bis*-SP derivatives with two styrylpyridinium moieties that are conjugated through the 1,3-trimethylene (propane) unit.

In this way, we attempted to modify the structure of novel putative fluorescent probes that also possessed anticancer activity to obtain theranostic properties. We designed fluorescent probes for the near-infrared (NIR) region comprising strong electron-donating moieties possessing one or three methoxy groups with a dimethylamino group or fixed dipropylamino group included in the julolidine moiety. We evaluated the possibility of modifying the absorption and emission wavelengths by obtaining a modified conjugated chain (incorporating one or two vinyl groups).

#### 2.1.2. Synthesis-Modified Conjugated Chain (Incorporating One or Two Vinyl Groups)

**Scheme 1:**
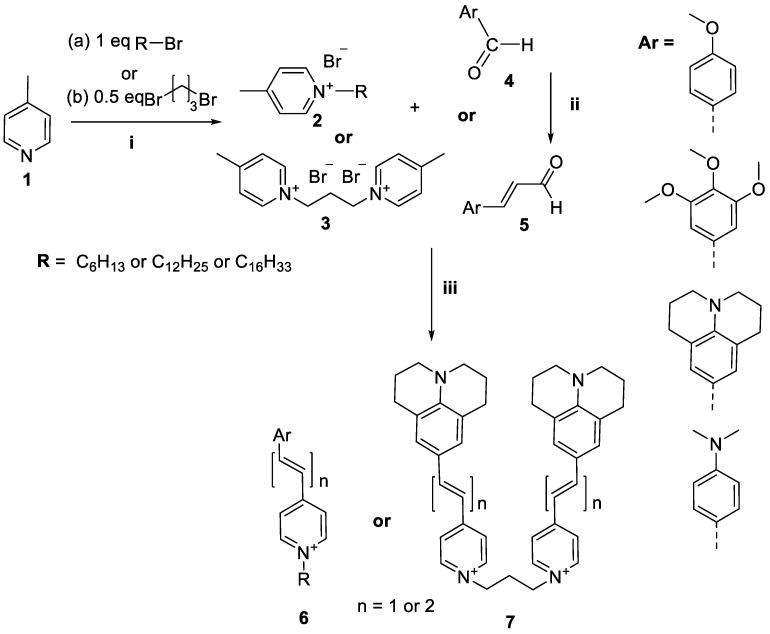
Schematic synthetic procedure of the proposed styryl derivatives: **i**: 2-butanone, 80 °C, 24 h; **ii**: in pressure vessel, 5 eq triphenyl (1,3-dioxolan-2-ylmethyl)phosphonium bromide, 4 eq NaH, and 0.12 eq 18-crown-6, dry THF, under Ar, RT, 2 h; **iii**: 2 eq aldehyde, 1 eq picolinium **2** or **3**, EtOH, three drops of piperidine, 90 °C, 24 h for compounds **6a**, **b**, **d**, **f**, **g**, and **7a** with vinyl linkers and in MeOH solution in the presence of 12.2 eq pyrrolidine at RT for 2 h for compounds **6c**, **e**, and **7b** with divinyl linkers (for details see Supplementary Materials).

The designed styrylpyridinium derivatives **6** and **7** were obtained from corresponding aldehydes **4** or **5** and 4-picolinium bromides **2** or *bis*-4-methylpyridinium dibromide **3** in EtOH solution in the presence of a catalytic amount of piperidine. For compounds with a vinyl linker, the reaction mixture was refluxed for 24 h in analogue using the procedures described by Vaitkiene et al. [8]. For compounds with a divinyl linker, synthesis was performed in MeOH solution in the presence of 12.2 eq pyrrolidine; the reaction mixture was stirred at RT for 2 h. The schematic synthetic procedures are presented in Scheme 1. The substituents for the structures of the obtained SP **6** and *bis*-SP **7** derivatives are depicted in Table 1.

The full detailed description of the synthesis and characterisation of the new styrylpyridines **6a**,**c**–**e** and their structural analogue *bis*-SP **7b** is given in the supplementary data (see Supplementary Materials). The structures of original SP **6a**,**c**–**e** and *bis*-SP **7b** were established and confirmed based on the ^1^H NMR, ^13^CNMR, and HRMS data (Figures S1–S15, Supplementary Materials). The data for SP derivatives **6b** [38], **6f** [39], **6g** [39], and *bis*-SP derivative **7a** [40] were in good agreement with the literature data. According to high-performance liquid chromatography (HPLC) data, the purity of the studied compounds was at least 97%.

### 2.2. Cytotoxicity

The evaluation of the in vitro cytotoxicity (IC**_50_**, half maximal inhibitory concentration) of the abovementioned compounds and their structural derivatives was assessed using the standard colorimetric 3-(4,5-dimethylthiazol-2-yl)-2,5-diphenyltetrazolium bromide (MTT) assay on two monolayer tumour cell lines, namely HT-1080 (human connective tissue fibrosarcoma) and MH-22A (mouse hepatocarcinoma). Additionally, the impact of the compound on non-cancerous “normal” cells—mouse fibroblasts (NIH3T3)—was estimated in order to measure the basal toxicity (LD_50_, median lethal dose). The obtained results were used for investigation of the structure–activity relationships and exploration of the effect of substituents on cellular response and toxicity. The results are presented in Table 2. The estimated LD_50_ gives information regarding the compound properties and could potentially be used to select promising compounds by providing a starting dose for in vivo experiments.

We determined the cytotoxicity of the styrylpyridinium compounds SP **6** and *bis*-SP **7** (Table 2). The characteristics of structure–property relationships were investigated by analysing the cytotoxicity towards cancerous cell lines (HT-1080, MH-22A) and basal toxicity (LD_50_) for the non-cancerous NIH3T3 cell line.

#### 2.2.1. The Effect of Alkyl Chain Length

The obtained data reveal that the compounds with long alkyl chains (C_12_H_25_ or C_16_H_33_) at the pyridinium nitrogen atom, in general, have high basal toxicity: from 249 mg/kg in the case of 4-(3,4,5-trimethoxystyryl)-*N*-dodecylpyridinium bromide (**6a**) (Table 2, **entry 1**), up to 85 mg/kg in the case of 4-methoxystyrylvinyl-*N*-hexadecylpyridinium bromide (**6c**) (Table 2, **entry 3**). As an exception, the LD_50_ = 477 mg/kg for 4-(julolidinyl-buta-1,3-dienyl-1)-*N*-hexadecyl pyridinium bromide (**6e**) (Table 2, **entry 5**) represented a medium LD_50_ value.

In the case of identical compounds with different lengths of alkyl chains (**6a** and **6b**), a longer alkyl chain increased the basal cytotoxicity from 249 to 91 mg/kg. Similar findings were observed in the case of **6f** and **6g**: the basal cytotoxicity increased from 413 to 203 mg/kg. Only in the case of **6d** and **6e** did a longer alkyl chain (in combination with divinyl linker) significantly decrease the basal cytotoxicity (from 169 to 477 mg/kg).

On the other hand, compounds possessing two styrylpyridinium or styrylvinylpyridinium (phenyldivinylpyridinium) moieties attached to a propane linker in positions 1 and 3 had significantly lower basal toxicity, 1102 mg/kg and 857 mg/kg, respectively (see compounds **7a** and **7b** (Table 2, **entries 8 and 9**)). These compounds can be regarded as geminal derivatives of **6d** and **6e**. Interestingly, the geminal compounds **7a** and **7b** were less toxic than **6d** and **6e** for all of the tested cell lines—both non-cancerous and cancerous ones.

#### 2.2.2. The Effect of the Electron-Donating (Ar) Group

For selected compounds, **6a**–**6f**, we performed a determination of the cytotoxicity in a wider variety of cancer and non-cancerous cell lines (see Table S2, Supplementary Materials) in order to learn the characteristic features of selectivity or the similarity of biological properties.

Compounds **6e** and **6c** are structurally identical except for the presence of different electron-donating groups. Replacing the julolidinyl group in compound **6e** with 4-metoxyphenyl moiety (compound **6c**) led to increased cytotoxicity and basal toxicity (Table 2, **entry 3**). The IC_50_ values decreased from 1 µg/mL to 0.2 µg/mL for the HT-1080 cell line and from 0.4 µg/mL to 0.13 µg/mL for the MH-22A cell line. The LD_50_ value decreased from 477 mg/kg to 85 mg/kg. Moreover, the impact was consistently observed across all tested cell lines, as outlined in the supplementary materials (Table S2, Supplementary Materials).

The insertion of a 3,4,5-trimethoxyphenyl moiety in compound **6a** instead of the julolidinyl derivative in compound **6d** moderately decreased the cytotoxicity. Comparing identical compounds (**6a** and **6d**) with different electron-donating groups, the cytotoxicity was lower for compound **6a**: 12.5 times lower for cell line BHK-21, 21.9 times lower for cell line NIH3T3, and 8.5 times lower for cell line MH-22A. Furthermore, the basal toxicity decreased by 1.5 times (see Table S2, Supplementary Materials).

Nevertheless, among all the tested cell lines, compound **6e** (with a julolidinyl electron-donating group) demonstrated the least toxicity when compared with the others.

#### 2.2.3. The Effect on Different Cell Lines

Interestingly, no significant similarities were found between the observed toxicities of the compounds on different cell lines. For example, the cytotoxicity of compound **6d** in the MH-22A cell line was very high (0.05 µg/mL) compared with the cell line HT-1080 (1.4 µg/mL). Additionally, compounds **6a** and **6b** had opposite comparative cytotoxicities in MH-22A (**6a** was 2.4 times more toxic) and basal toxicities (compound **6b** was 2.8 times more toxic). These findings suggest that the cytotoxic effects of the compounds are influenced by factors specific to each cell line rather than because of a common biological pattern. Thus, the cytotoxicity of these compounds cannot be easily predicted or generalised. Instead of cytotoxic promiscuity, there seems to be a level of specificity, indicating that the compounds may interact with distinct cellular targets or pathways in a cell-line-dependent manner. Similar observations have been previously demonstrated in the literature [41,42,43].

Certain compounds exhibited enhanced cytotoxicity against cancer cells. For instance, compound **6d** had 6.5 times higher basal cytotoxicity than compound **7a** and 80 times higher cytotoxicity towards the cell line MH-22A than compound **7a** (Table 2, **entry 4**). Therefore, the SP compound **6d** could represent a potential anticancer drug as it possesses cancer-cell-specific toxicity. Similarly, it was found that compound **6e** has high and comparatively selective cytotoxicity towards the MH-22A (mouse hepatocarcinoma) cell line: IC_50_ = 0.4 µg/mL. This is 3.75 times more toxic than against the non-cancerous Hek-293 (human embryonic kidney cells) cell line and eight times more cytotoxic than against the non-cancerous NIH3T3 cell line. If we compare identical compounds with varying lengths of the alkyl chain, i.e., **6d** and **6e**, the basal cytotoxicity was ~2.8 times lower in the case of **6e**, whereas the cytotoxicity towards the HT-1080 cell line was 1.4 times higher.

The obtained data support the selection of specific compounds for further in-depth studies. Practically, compounds **6e**, **7a**, and **7b** possesses the lowest cytotoxicity towards non-cancerous cell lines, making them attractive candidates for further investigation or development. Remarkably, the SP compound **6e** has moderate cytotoxicity towards non-cancerous cell lines and more expressive cytotoxicity towards several cancer cell lines (e.g., MH-22A).

### 2.3. Fluorescence

**Figure 2:**
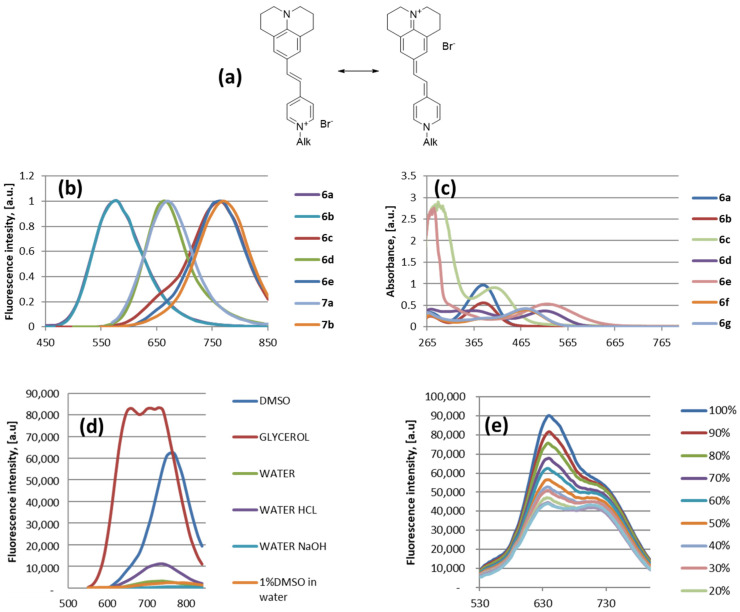
The spectroscopic properties of compounds **6a**–**e** and **7a**,**b**: (**a**) resonance structures of compounds **6d**,**e**; (**b**) normalised fluorescence spectra of compounds **6a**–**e** and **7a**,**b** in DMSO solution; (**c**) UV/Vis spectra of compounds **6a**–**g** in DMSO, the concentration for compounds **6a**,**b**,**d**,**f**,**g** is 0.33 mM and for compounds **6c**,**e** is 5 mM; (**d**) fluorescence spectra of compound **6e** in various media: DMSO (0.5 mM); glycerol (0.01 mM); water (0.5 mM); 0.1 M HCl (0.5 mM); 0.1 M NaOH (0.5 mM); and 1% DMSO/99% water (5 mM); (**e**) fluorescence spectra of compound **6e** (5 mM) in EtOH–glycerol mixtures (0%—pure EtOH:100%—pure glycerol).

The spectroscopic properties of compounds **6a**–**e** and **7a**,**b** were studied, and the results are presented in Figure 2 and Table 3. The resonance structures of compounds **6d**,**e** (Figure 2a) show a possibly intramolecular charge transfer between the julolidine moiety and the pyridinium unit. The normalised fluorescence spectra of the tested compounds **6a**–**e** and **7a**,**b** in DMSO solution (Figure 2c) and the fluorescence spectra of compound **6e** in various media (Figure 2d) were measured using a spectrophotometer multiplate reader (Infinite M1000). The UV/Vis spectra in DMSO solution of compounds **6a**–**g** (Figure 2b) were reordered with a UV/Vis spectrophotometer, and the fluorescence spectra of compound **6e** in EtOH–glycerol mixtures (Figure 2e) were collected with a Spectrofluorometer FS5.

It was found that the maximum fluorescence intensity was observed when the samples were excited at 380 nm for compounds **6a**,**b** and at 530 nm for compounds **6c**–**e** and **7a**,**b**. Compounds **6a**,**b**, with 3,4,5-trimetoxyphenyl moieties and vinyl linkers, had a fluorescence maximum at 580 nm, whereas SP **6c**, with a 4-metoxyphenyl moiety and a divinyl linker had a fluorescence maximum at 760 nm. Compounds with julolidinyl groups and vinyl linkers (SP **6d** and *bis*-SP **7a**) showed a fluorescence maximum at 660 nm, whereas compounds with a divinyl linker (SP **6e** and *bis*-SP **7b**) showed fluorescence maximums at 760 nm and 770 nm, respectively (Figure 2b, Table 3, **entry 1**). The theoretical calculations using the TDDFT method (the details are given in Supplementary Materials) revealed that the excitation and emission mechanism for SP compound **6e** follows the mechanism proposed by Jablonski [44]. According to the calculations, the excitation occurs at 542 nm and the emission at 765 nm. The obtained theoretical calculation data are in good agreement with the experimental data, where the excitation occurs at 530 nm and the emission at 760 nm (Table 3).

We have also tested whether the characteristics of compound **6e** depended upon the viscosity of its surrounding environment. The fluorescence intensity dramatically increased in viscous media such as glycerol, but it was lower in water media and basic or acidic media, as well as in 1% DMSO solution in water as a mimic of in vivo conditions (Figure 2d). It was underlined that water media reduced the fluorescence quantum yields and the lifetime of organic fluorophores by up to threefold [45]. We also demonstrated fluorescence intensity changes for SP compound **6e** with different ethanol/glycerol ratios to show the viscosity-dependent results (Figure 2e). In pure glycerol, the maximum value of fluorescence was detected at 645 nm. As the amount of glycerol decreased, the fluorescence intensity of the sample decreased as well. The second fluorescence maximum at 720 nm was observed when the amount of ethanol had reached approximately 50% in the composition.

The spectral properties (absorption and fluorescence maximum in DMSO solution) of compounds **6a**–**e** and **7a**,**b**, UV spectral data (extinction coefficient (log ε) and absorption maximum), values of Stokes’ shift, and fluorescence quantum yield are presented in Table 3.

The studied compounds have large Stokes’ shift values (Table 3, **entry 3**)—around 195 nm for compounds **6a** and **b**; 240 nm for compound **6e**; 325 and 352 nm for compounds **6d** and **6c**, respectively. The revealed properties of the reported fluorescent compounds are in line with the results of recent articles on NIR styryl dyes [46], where it is indicated that very large Stokes’ shifts are crucial in bioimaging applications. Synthesis and studies of styryl dyes are continuing with the purpose of searching for efficient plasma membrane probes [47], highly selective fluorescent probes for lysosome visualisation in live cells [48], probes to visualise the nucleus in live cells [49], and probes for mitochondria imaging in live cells [50].

Photoluminescence quantum yield (PLQY) values for the DMSO solution of compounds **6a**–**g** were measured (Table 3, **entry 4**). The highest PLQY values were observed for the SP compounds **6a**,**b** (each with a 3,4,5-trimetoxyphenyl moiety), which were 15.1% and 12.2%, respectively. The PLQY values for the SP derivatives **6f**,**g** (each with an *N*-dimethylaminophenyl moiety) were 1.6% and 1.4%, respectively. The PLQY value for the SP derivative **6c** (with a 4-dimethylaminophenyl moiety) was only 0.1%. The PLQY values for the SP derivatives **6d**,**e** (each with a julolidinyl moiety) were 0.5% and 0.7%, respectively. Previously, we designed naphthalene-2-yl)vinyl)pyridinium groups containing 1,4-dihydropyridine amphiphile, for which the PLQY value for the EtOH solution was 1% [20].

Thus, after analysis of the data regarding absorption and fluorescence maximum for the compounds, we proposed that the SP compounds **6c**,**e** and *bis*-SP **7b** have a useful pleiotropic combination of putative diagnostic and chemotherapeutic, i.e., theranostic, properties.

### 2.4. Self-Assembling Properties

Compounds with a cationic moiety and long alkyl chains in the molecule could possess self-assembling properties. The estimation of self-assembling properties for the selected compounds was performed using dynamic light scattering (DLS) measurements. The values for the polydispersity index (PDI) and the hydrodynamic average diameter (D_av_) of nanoparticles formed by compounds **6a**,**c**–**e**,**g** were determined (with a final compound concentration of 0.1 mM in the aqueous medium). The nanoparticles were prepared by the ethanol injection method via the dissolution of the compound in ethanol and after that by the dispersion of the solution into water. The approach is rapid, simple, and can be easily scaled up [51]. This method was previously used successfully for the preparation and characterisation of lipid-like pyridinium amphiphiles [20,52,53]. The obtained results are summarised in Table 4. The DLS measurements were performed for freshly prepared samples at room temperature.

The obtained data demonstrate that all of the samples were sufficiently homogeneous, with PDI values in the range 0.218 to 0.331. Compound **6a** formed larger particles with an average diameter of around 600 nm, whereas for compound **6d** the particle diameter was around 350 nm. The other tested compounds, **6c**, **e**, and **g**, formed smaller nanoparticles with average diameters of 212, 118, and 207 nm, respectively. In the case of *bis*-SP **7**, we obtained unanalysable samples, probably due to the insufficient lipophilic characteristics of the compound molecule.

We analysed the obtained data regarding compound cytotoxicity, fluorescence, and self-assembly properties and selected compound **6e** for the detailed cell labelling studies. Compound **6e** possessed the lowest cytotoxicity towards non-cancerous cell lines and more expressive cytotoxicity towards several cancer cell lines, demonstrated a large Stokes’ shift, and had self-assembling properties. By combining therapeutic and diagnostic functionalities, this dual-purpose compound demonstrates its theranostic capabilities. Theranostic approaches allow for precise and targeted therapy based on the specific characteristics of a patient’s disease, while also enabling the ability to monitor the response to treatment and make adjustments if needed [54,55,56,57,58].

### 2.5. SP Compound ***6e*** for Cell Staining

SP compound **6e** was selected for fluorescent cell imaging analysis. First, the compound was used to label the BHK-21 cells that are widely used in molecular biology studies [59,60,61]. Commercially available CellTracker™ Deep Red dye (Invitrogen) was used as a control because it has a similar emission range (ex/em: 630/660). The BHK-21 cells were incubated with fluorescent dyes to stain the cells, then seeded on a 24-well plate and cultured for 24 h. SP compound **6e** was used at 25 μM concentration, providing a non-toxic effect on BHK-21 cells (Table S2, **entry 5**, Supplementary Materials). The Deep Red dye was used according to the manufacturer’s instructions.

**Figure 3:**
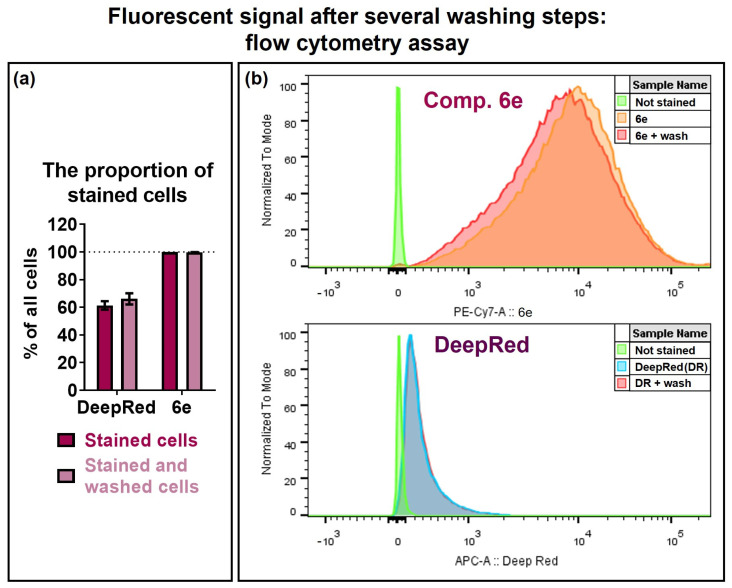
Fluorescence and stability of the fluorescent signal in BHK-21 cells stained with SP compound **6e** in comparison with the commercially available dye analogue CellTracker™ Deep Red (Cat. No. C34565; Invitrogen, Waltham, MA, USA). BHK-21 cells were treated with 1 μM Deep Red or 25 μM compound **6e** for 15 min. The cells were washed and cultured for 24 h in a fresh medium, after which the cells were collected and washed again 3 times with PBS (+wash) or analysed without washing. The number of fluorescent cells was quantified by flow cytometry. Unstained BHK-21 cells were used as a control. (**a**) The proportion of stained cells. Data are presented as mean ± SEM (*n* = 3). (**b**) Fluorescence intensity is presented as histograms of stained cells in respective channels.

The day after staining, the cells were collected and washed several times with PBS buffer in order to evaluate the stability of the staining. The fluorescent signal was analysed by flow cytometry (Figure 3). Both compound **6e** and the Deep Red dye exhibited relatively high stability, as the fluorescent signals of both dyes remained unchanged after cell washing. However, only (61 ± 3)% of Deep Red stained cells were positive, whereas SP compound **6e** ensured 99.9% positive cells. Moreover, the signal intensity (fluorescence shift from unstained cells) of cells stained with compound **6e** was remarkably higher than for the Deep Red dye. The calculated stain index for SP compound **6e** was (92 ± 19) before cell washing and (77 ± 15) after cell washing. However, in the case of Deep Red, the calculated stain index was (2.27 ± 0.07) before cell washing and (2.21 ± 0.07) after cell washing. High intensity usually facilitates flow cytometry data acquisition by enhancing the signal-to-background ratio and enabling optimal compensation in complex multicolour fluorescence analysis.

**Figure 4:**
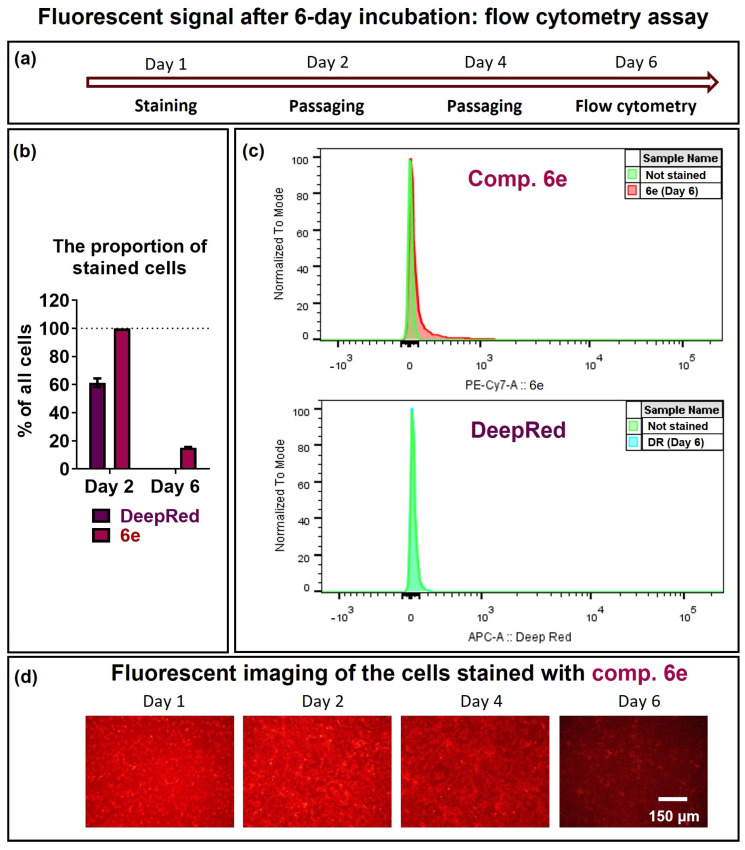
Fluorescence and stability of the fluorescent signal in BHK-21 cells stained with compound **6e** in comparison with the commercially available dye analogue CellTracker™ Deep Red (Cat. No. C34565; Invitrogen). BHK-21 cells were treated with 1 μM Deep Red or 25 μM compound **6e** for 15 min and washed with PBS. Then, the cells were cultured in a fresh media for six days (passaged twice), collected on day 6, and the % of fluorescent cells was measured by flow cytometry. (**a**) The timeline of the experimental design. (**b**) The proportion of stained cells. Data are presented as mean ± SEM (*n* = 3). (**c**) Fluorescence intensity is presented as histograms of stained cells in respective channels. (**d**) The cell fluorescence microscopy at indicated days after staining is shown for compound **6e**. The fluorescent imaging was performed using a Leica DM-IL fluorescent microscope with an N2.1 filter (excitation: 515–560 nm, emission: 580 nm).

In order to assess the stability of staining after prolonged cell culture, the stained BHK-21 cells (Day 1) were passaged twice (Days 2 and 4) and cultured for six days in total (Day 6, Figure 4). On day 6, the cells were collected and analysed by flow cytometry. The number of positive fluorescent cells decreased significantly: no positive cells were detected in the Deep Red sample, whereas (15.0 ± 0.7)% were detected in the SP-compound-**6e**-stained sample. The cells stained with SP compound **6e** were visualised by fluorescent microscopy, which showed the decrease in the red fluorescence signal on day 6 compared with day 1, demonstrating a low but detectable level for the positive cells (Figure 4). It can be concluded that, in contrast to the Deep Red cell tracker, SP compound **6e** effectively stains cells and can be used for up to 6 days of cell culture.

### 2.6. Staining of Primary Cells

**Figure 5:**
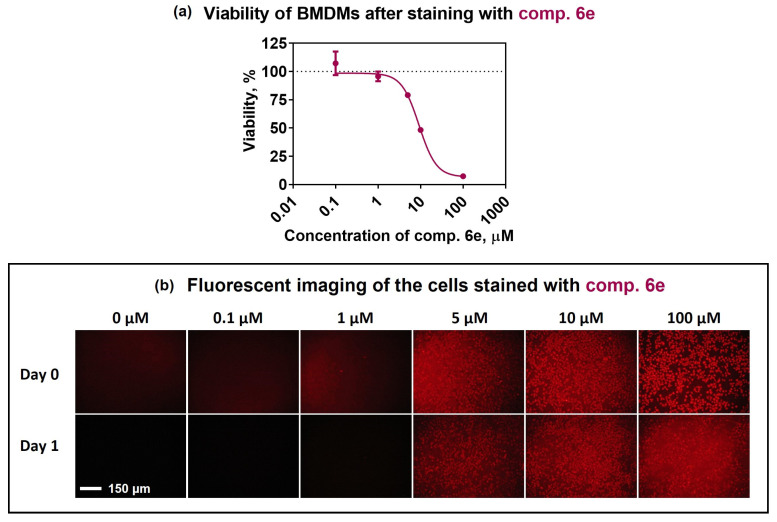
CCK-8 viability assay and fluorescence microscopy of BMDMs stained with SP compound **6e** at different concentrations. BMDMs were incubated in a medium supplemented with compound **6e** for 15 min at 37 °C, after which they washed with PBS and incubated overnight in a fresh media. (**a**) Viability of the BMDMs stained with compound **6e** at different concentrations. Data are presented as mean ± SEM (*n* = 3). (**b**) Fluorescent microscopy pictures were captured using a Leica DM-IL fluorescent microscope with an N2.1 filter (excitation: 515–560 nm, emission: 580 nm) immediately after staining (Day 0) and after the overnight incubation (Day 1).

Primary cells, unlike many cancerous cell lines, undergo senescence processes and have limited potential for self-renewal. Primary immune cells are very sensitive to culture conditions and their staining is challenging due to the toxicity of dyes. In this study, we used murine bone-marrow-derived macrophages (BMDMs) to evaluate the staining potential of the 6e compound in primary immune cells. BMDMs represent immune cells with high plasticity that are used in many human disease-related studies, including those involving autoimmune diseases and cancer [62,63,64]. The first step was to examine whether SP compound **6e** has an impact on cell viability. Bone-marrow-derived macrophages are progenitors that are able to differentiate into pro-inflammatory M1 and anti-inflammatory M2 phenotypes, which ideally fit to demonstrate the functional activity of labelled cells in routine biological studies [65,66,67,68,69]. To assess toxicity, the BMDMs were stained with various concentrations (0.1–100 μM) of compound **6e**, and after 24 h, cell viability was measured using the CCK-8 assay (Figure 5). The calculated IC_50_ value was found to be (9.2 ± 1.5) μM; however, according to the fluorescence microscopy images, the staining was sufficient at a 5 μM concentration, corresponding to the rather high proportion (79.1 ± 1.2)% of viable cells.

**Figure 6:**
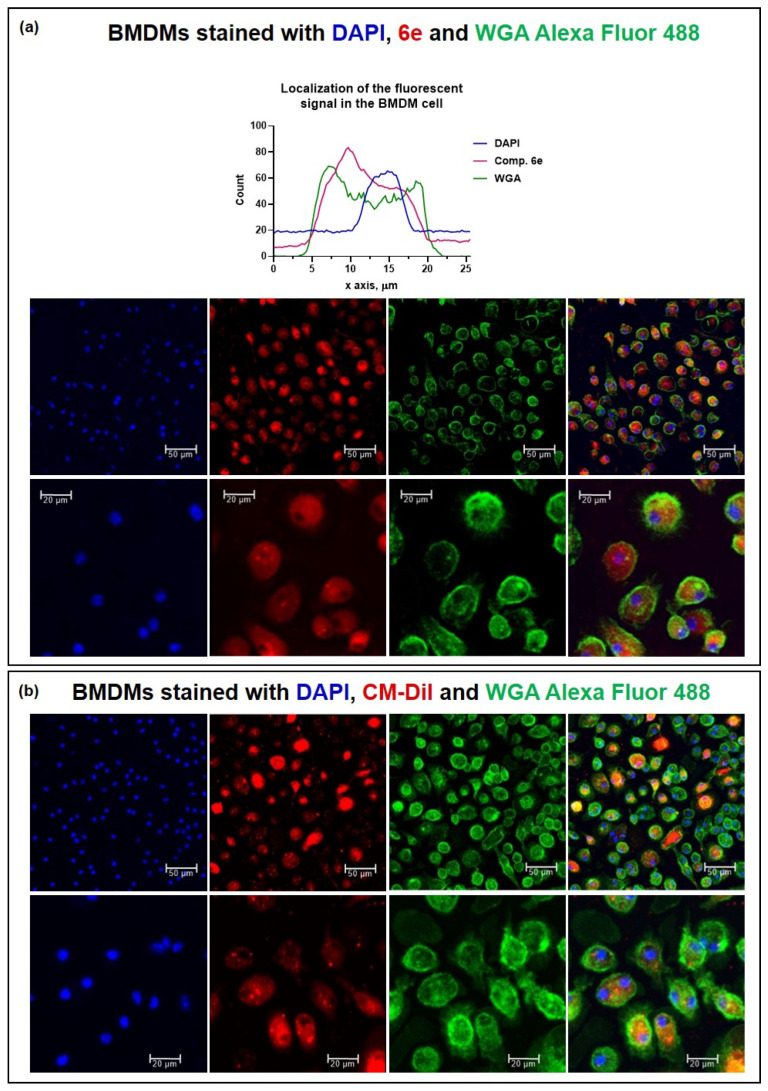
The stained BMDMs were studied using confocal laser scanning microscopy with a Leica TCS SP8. A DPSS (561 nm) laser was used for the excitation of compound **6e** and CM-Dil, an argon (488 nm) laser for WGA, and a diode (405 nm) laser for DAPI. For fluorescence detection, HyD was used for compound **6e** and CM-Dil (578 nm–636 nm) and PMT detectors were used for WGA (493 nm–739 nm) and DAPI (417 nm–498 nm), pinhole at 95 µm. (**a**) Confocal microscopy and localisation of the fluorescent signal of BMDMs stained with DAPI, 5 μM compound **6e**, and 1 μg/mL WGA Alexa Fluor 488. (**b**) Confocal microscopy of BMDMs stained with DAPI, 4 μM CM-DiI, and 1 μg/mL WGA Alexa Fluor 488.

The stained BMDMs were studied using confocal microscopy to verify the membrane, cytoplasmic, or nuclear localisation of the staining (Figure 6). Wheat germ agglutinin (WGA) Alexa Fluor 488 (Invitrogen, W11261) was chosen for selective plasma membrane labelling and DAPI (Invitrogen, D1306) was selected for nucleus labelling. It was found that the dye **6e** was localised in the cell cytoplasm and does not stain the cell nucleus (Figure 6a). Furthermore, the SP compound **6e** was compared with commercially available CM-DiI Dye (Invitrogen, ex/em: 553/570, Figure 6b).

The staining with compound **6e** appeared to be more evenly distributed and the level of noise was lower. These observations suggest that compound **6e** offers advantages in terms of achieving a clearer signal-to-noise ratio and better overall imaging quality. The homogeneous cytoplasmic distribution and lower noise level obtained with compound **6e** staining contribute to improved visualisation and accurate analysis of the sample, enhancing the reliability of data.

**Figure 7:**
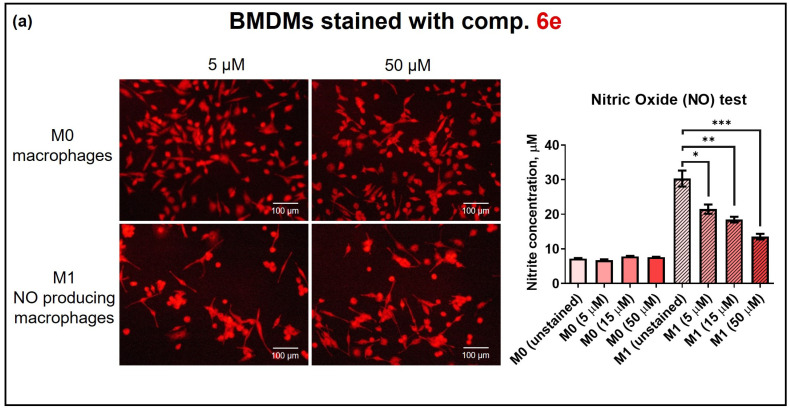

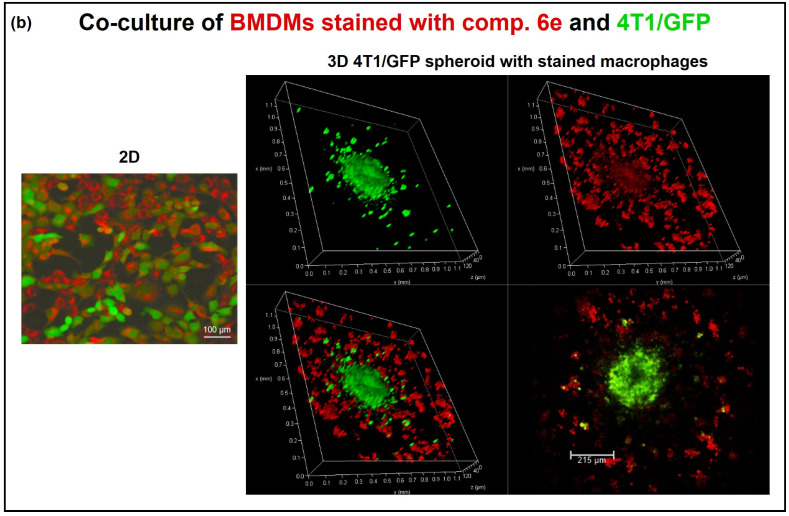
Activation of pre-stained macrophages to the M1 phenotype and co-cultivation with cancer cell spheroids. (**a**) Bone-marrow-derived macrophages (M0) were stained with compound **6e** at various concentrations (5, 15, and 50 μM) and used for fluorescent microscopy imaging. A DPSS (561 nm) laser was used for the excitation of compound **6e** and HyD was used for fluorescence detection. The level of nitric oxide (NO) was determined in cell culture supernatants after 2 days of BMDM activation with IFNγ/Pam3SCK4 ligand. The bars represent the mean values ± SEM. * *p* = 0.0292; ** *p* = 0.0083; *** *p* = 0.0023; *n* = 3. (**b**) Confocal microscopy of BMDMs stained with 5 μM compound **6e** co-cultivated with 4T1 mammary cancer cells expressing GFP (green): (left) monolayer; (right) 3D culture. A DPSS (561 nm) laser was used for the excitation of compound **6e** and an argon (488 nm) laser for GFP. For fluorescence detection, HyD was used for compound **6e** and a PMT detector was used for GFP (493–560 nm), pinhole at 95 µm.

The impact of SP compound **6e** on the BMDMs’ functional characteristics was also assessed by performing cell differentiation into the M1 phenotype in the presence of compound **6e**. The BMDMs were treated with 5–50 μM compound **6e** and the cell medium was replaced with a macrophage differentiation medium containing recombinant mouse interferon-gamma (IFNγ) and the TLR2/1 ligand PAM3SCK4 to ensure M1 polarisation [37,65]. Activated cells express proinflammatory molecules (cytokines and chemokines), as well as nitric oxide (NO) as the main features of their proinflammatory M1-like macrophage phenotype. To verify the NO level in cell culture media, nitrites, as nitric oxide oxidation products, were measured by the standard Griess test. The nitric oxide assay demonstrated that BMDMs stained with compound **6e** displayed strong NO production after treatment with IFNγ and PAM3SCK4 (Figure 7a). Although, the maximal NO production was observed for unstained cells (30 ± 2 µM), the stained BMDMs also revealed relatively high NO production (up to 21.5 µM). However, as the amount of compound 6e increased, the NO signal gradually decreased. These results strongly suggest a correlation with a reduction in cell viability, as depicted in Figure 5a. The standard fluorescence microscopy analysis demonstrated a specific and bright red fluorescence signal in stained BMDM cells at less toxic concentrations of compound **6e** (5 µM). We concluded that stained M1 macrophages produce NO; therefore, the pre-stained BMDMs can be used for functional programming, as well as for cell tracking and cell migration assays.

Since the macrophages have a significant role in cancer progression, the co-cultivation of **6e**-stained BMDMs with murine mammary cancer cells expressing green fluorescent protein (4T1/GFP) was tested. In summary, the experiment involved staining BMDMs with compound **6e** and introducing them to 4T1/GFP cells cultured in a monolayer. The staining facilitated cell identification in co-culture (Figure 7b). Next, a cancer cell spheroid was formed by incubating 4T1/GFP cells in an ultra-low attachment plate. The pre-stained BMDMs were then added to the cancer cell spheroid, and the sample was analysed using confocal microscopy. The confocal microscopy revealed interactions between the cancer cell spheroid and macrophages. Remarkably, macrophage-induced cancer cell migration out of the spheroid could be detected by the co-localisation of BMDMs and cancer cells.

By employing pre-stained BMDMs in conjunction with cancer cell spheroids, researchers can gain valuable insights into the interactions between cancer cells and macrophages, shedding light on the processes of cell migration and phagocytosis in the context of cancer progression. The confocal imaging data clearly show the high potential of the **6e** probe for use in the imaging of co-cultures (BMDMs + 4T1/GFP) and the investigation of cell–cell interactions in both 2D and 3D cancer cell models [37,70] (Figure 7b).

## 3. Materials and Methods

### 3.1. Chemistry

More detailed descriptions of the synthetic procedures and characterisation of the original unpublished intermediates and compounds are described in the Supplementary Materials.

### 3.2. Cell Culture and Measurement of Cell Viability

The HT-1080 (human connective tissue fibrosarcoma, ATCC^®^ CCL-121™), MH-22A (mouse hepatocarcinoma, ECACC Cat. No. 96121721), A-549 (human lung carcinoma, ATCC^®^ CCL-185™), NIH3T3 (Swiss Albino mouse embryo fibroblast, ECACC Cat. No. 93061524), Hek293 (human embryonic kidney, ATCC^®^ CRL-1573™), BHK-21 (Syrian hamster kidney, ATCC^®^ CCL-10™), A-431 (human epidermoid carcinoma, ATCC^®^ CRL-1555™), Saos-2 (human osteosarcoma, ATCC^®^ HTB-85™), HeLa (human epitheloid cervix carcinoma, ATCC^®^ CCL-2™), U937 (human myeloid leukaemia, ATCC^®^ CRL-1593.2™), C6 (rat glioma, ATCC^®^ CCL-107™), and B16-F10 (murine melanoma, ATCC^®^ CRL-6475™) cell lines were obtained from the American Type Culture Collection (ATCC) and European Collection of Authenticated Cell Cultures (ECACC).

HT-1080, MH-22A, A-549, NIH3T3, BHK-21, A-431, HeLa, and B16-F10 cells were cultured in Dulbecco’s Modified Eagle’s Medium (DMEM) with 2 mM glutamine and 10% foetal bovine serum (FBS). Hek293 cells were cultured in Eagle’s Minimum Essential Medium with 10% foetal bovine serum. U937 cells were cultured in RPMI-1640 Medium with 2 mM glutamine and 10% foetal bovine serum. C6 cells were cultured in Ham’s F12 Medium with 2 mM glutamine and 10% foetal bovine serum. Saos-2 cells were cultured in McCoy’s 5a Medium with 2 mM glutamine and 10% foetal bovine serum. The cells were incubated at 37 °C with 5% CO_2_, 95% air, and complete humidity. Once ~90% confluence was reached, they were detached using 0.05% trypsin/EDTA and counted. The cells were plated at optimally determined densities for the logarithmic phase of growth. These cells were then suspended at a concentration of 4000 cells/well for HT-1080, MH-22A, A-549, NIH3T3, BHK-21, A-431, HeLa, B16-F10, Hek293, and Saos-2 cells and a concentration of 9000 cells/well for U937, C6, Hek293, and GM08402 cells and added onto a 96-well plate. For background absorption, some wells remained cell free, i.e., as a blank control. Cells were incubated for 72 h at 37 °C and 5% CO_2_ where they were exposed to a media containing serial dilutions of the investigated compounds (*n* = 3). Cell viability was measured using the 3-(4,5-dimethylthiazol-2-yl)-2,5-diphenyl-tetrazolium bromide (MTT) assay [71]. In brief, after incubating with compounds, the culture medium was removed and fresh medium with 0.2 mg/mL MTT was added to each well of the plate. After incubation (3 h, 37 °C, 5% CO_2_), the medium with MTT solution was removed and replaced with 200 µL DMSO and 25 µL Sorenson’s glycine buffer (glycine 0.1 M, NaCl 0.1 M, pH: 10.5 with 0.1 M NaOH). The plate was shaken for 15 min at room temperature and the optical density (OD) of the wells was determined using a multichannel spectrophotometer (Thermo Scientific Multiskan EX) at 540 nm. The quantity of the control cells was taken in calculations as 100%. The IC_50_ value was calculated using the program Graph Pad Prism^®^ 3.0.

### 3.3. Evaluation of Basal Cytotoxicity

To assess basal cytotoxicity, the neutral red uptake (NRU) assay was conducted following a modified version of the standard protocol of Stokes’ [71], as validated by the NICEATM-ECVAM study [72]. The NRU cytotoxicity assay determines the ability of viable cells to incorporate and bind a supravital dye, neutral red (NR).

NIH3T3 cells (9000 cells/well) were cultured in 96-well plates in DMEM medium supplemented with 5% FBS for 24 h. Subsequently, the cells were exposed to the test compounds over a range of seven concentrations (1000 µg/mL, 316 µg/mL, 100 µg/mL, 31 µg/mL, 10 µg/mL, 3.0 µg/mL, and 1.0 µg/mL) for 24 h. Untreated cells were used as a control. After the incubation period, the medium was removed from all plates and a solution of NR was added (250 µL, 0.05 mg/mL NR in DMEM 24 h pre-incubated at 37 °C and then filtered before use through a 0.22 µm syringe filter). The plates were incubated for 3 h, followed by three washes with phosphate-buffered saline (PBS). The cells were then treated with a mixture of ethanol (50%) and water (49%) supplemented with 1% acetic acid. The absorbance of NR was measured using a spectrophotometer multiplate reader (Infinite M1000; Tecan Austria GmbH, Salzburg, Austria) at 540 nm. The optical density (OD) was then calculated using the following formula:

$$\frac{{OD}_{treated\;cells}\times 100}{{OD}_{control\;cells}}.$$

The IC_50_ values were calculated using the program Graph Pad Prism^®^ 3.0.

### 3.4. Estimation of LD_50_ from IC_50_ Values

In vitro test results were employed to estimate the starting dose for acute oral systemic toxicity tests in rodents (LD_50_). The LD_50_ value [29,73] was calculated using the in vitro IC_50_ value and a following formula:

$$log{LD}_{50}(mM/kg)=0.439\times log{IC}_{50}(mM)+0.621$$

The value was then converted to mg/kg and the compounds classified into four toxicity categories [74]. Category 1 (highly toxic): LD_50_ ≤ 5 mg/kg; Category 2 (moderately toxic): 5 < LD_50_ ≤ 50 mg/kg; Category 3 (slightly toxic): 50 < LD_50_ ≤ 300 mg/kg; Category 4 (practically non-toxic): 300 < LD_50_ ≤ 2000 mg/kg. By employing an alternative in vitro method, it becomes possible to compare the potential toxicity of new compounds and select candidates for further study, significantly reducing the number of animal experiments.

### 3.5. Excitation/Emission Fluorescence Spectra Measurements

A concentration of 10 mM stock solution of compounds **6** or **7** was prepared by dissolving the compounds in DMSO. Working solutions were prepared by diluting the 10 mM stock solution of compounds with different solvents to reach the final concentration for the samples: DMSO (compound concentration 0.1 mM); for compound **6e**: in glycerol (concentration 0.01 mM), in water (concentration 0.5 mM), in 0.1 M HCl (concentration 0.5 mM), in 0.1 M NaOH (concentration 0.5 mM), or in 1% DMSO/99% water (concentration 5 mM). The fluorescence spectra were measured using a spectrophotometer multiplate reader (Tecan Infinite M1000; Tecan Austria GmbH, Salzburg, Austria). For excitation/emission fluorescence spectra measurements, the following measurement parameters were applied: excitation wavelength from 260 nm to 690 nm with a 10 nm sampling interval and emission wavelength from 300 nm to 840 nm with a 10 nm sampling interval. The fluorescence spectra of compound **6e** (concentration 5 mM) in EtOH–glycerol mixtures were measured with an Edinburgh Instruments FS5 Spectrofluorometer (Edinburgh Instruments Ltd., Kirkton Campus, UK). The data were analysed using Microsoft Excel software version 2308.

### 3.6. Assessment of the Self-Assembling Properties of Compounds ***6*** and ***7*** Using Dynamic Light Scattering (DLS) Measurements

The self-assembling properties of the tested compounds were estimated according to a published procedure with minor modifications [52,53]. Stock solutions of the compounds were prepared in EtOH at a concentration of 1 mM. Stock solutions of the compounds (0.3 mL) were injected into 2.7 mL of deionised water with maximum stirring (IKA Vortex 2 (IKA, Staufen, Germany)) to obtain a final concentration of 0.1 mM. The DLS measurements of the nanoparticles in the aqueous solution were conducted using a Zetasizer Nano ZSP (Malvern Panalytical Ltd., Malvern, UK) instrument with Malvern Instruments Ltd. Software 8.01.4906. The measurements were performed with the following settings—medium: water; refractive index: 1.33; viscosity: 0.8872 cP; temperature: 25 °C; dielectric constant: 78.5; nanoparticles: liposomes; refractive index of materials: 1.60; detection angle: 173° wavelength: 633 nm. The obtained data were analysed using the multimodal number distribution software provided with the instrument. To ensure reproducibility, the measurements were performed in triplicate.

### 3.7. Stability of Dye during Cell Washing Steps

BHK-21 cells were plated onto a 6-well plate and cultured in Glasgow’s MEM (Cat. No. 21710025; Gibco, Life Technologies, Carlsbad, CA, USA) supplemented with 5% FBS, 10% tryptose phosphate broth solution (Cat. No. 18050039; Gibco, Life Technologies), 20 mM HEPES (Cat. No. 15630056; Gibco, Life Technologies), 2 mM L-glutamine (Cat. No. 25030024; Gibco, Life Technologies), 50 U/mL penicillin, and 50 mg/mL streptomycin (1% PEST; Cat. No. 15070063; Gibco, Life Technologies).

When cells reached 90% confluency, they were stained with 25 μM compound **6e** or 1 μM CellTracker™ Deep Red dye (Cat. No. C34565; Invitrogen) according to the manufacturer’s instructions. Briefly, the 10 mM stock solution of SP compound **6e** was prepared in ethanol and stored at 4 °C in the dark. Then, the dye was dissolved in DMEM (Cat. No. 31966047; Gibco, Life Technologies) and the cells incubated with the dye solution in DMEM for 20 min at 37 °C. Then, the dye solution was replaced with complete medium. A total of 24 h after staining, the cells were collected for flow cytometry by trypsinisation using 0.05% trypsin solution (Cat. No. 15400054; Gibco, Life Technologies) and washed three times with PBS supplemented with 2–10% FBS. Stained cells were kept at 4 °C and analysed using a FACSAria BD Hardware flow cytometer with FACSDiva 6.1.3 software (BD Biosciences, San Jose, CA, USA). A 488 nm blue laser and 780/60 bandpass filter (PE-Cy7) were used to analyse the SP compound **6e** signal; a 633 nm red laser and 660/20 bandpass filter (APC) were used to analyse the Deep Red signal. The data were analysed using FlowJo 10.3 software (FlowJo LLC, Ashland, OR, USA) and presented as the mean of three independent experiments. The stain index was calculated using following equation:

$$\frac{{Median}_{Positive}-{Median}_{Negative}}{2\times {SD}_{Negative}}$$

where ${Median}_{Positive}$ is the median intensity value of the positive population, ${Median}_{Negative}$ is the median intensity value of the negative population, and ${SD}_{Negative}$ is the standard deviation of the negative population intensity value.

### 3.8. Cell Passaging Assay

For the cell passaging, on day 0, BHK-21 cells were plated on a 6-well plate with a seeding density of 1.5 × 10^5^ cells per well. On day 1, the cells were stained with 25 μM compound **6e** or 1 μM CellTracker™ Deep Red dye as described above. The cells were incubated in a humidified 5% CO_2_ incubator at 37 °C and passaged on day 2 and day 4 using 0.05% trypsin solution. The fluorescent imaging was performed using a Leica DM-IL fluorescent microscope with N2.1 filter (excitation: 515–560 nm, emission: 580 nm). On day 6, cells were collected for flow cytometry analysis. The cells were kept at 4 °C and analysed as described above.

### 3.9. Isolation of Bone-Marrow-Derived Macrophages (BMDMs)

Mouse macrophages were derived from bone marrow cells following an established protocol employing the L929 cell-conditioned medium (CM) as a source of macrophage colony-stimulating factor (M-CSF) [65,66].

In summary, bone marrows were extracted from the femurs and tibiae of female BALB/c mice (8–12 weeks old). The bone marrow cells were flushed with RPMI-1640 medium supplemented with 10% FBS under sterile conditions and filtered through a 70 µm cell strainer (Cat. No. 800070; Bioswisstec, Schaffhausen, Switzerland). ACK lysis buffer (Cat. No. A10492-01; Gibco, Life Technologies) was employed to remove red blood cells via a 10 min incubation at room temperature. The remaining cells were then cultured in 10 cm non-tissue-culture-treated dishes (Cat. No. 0030702018; Eppendorf, Hamburg, Germany) in RPMI-1640 medium containing 30% L929 CM. The isolated bone marrow cells were cultured for 5 days, after which non-adherent cells were washed off with PBS. The adherent BMDMs were cultured for 24 h and then detached by incubation in PBS for 30 min at 4 °C followed by gentle flushing of the dish. The BMDMs were collected by centrifugation, frozen in Recovery™ Cell Culture Freezing Medium (Cat. No. 12648010; Gibco, New York, NY, USA), and stored at −150 °C for future experiments. Afer thawing, BMDMs were incubated for 4 days, detached, filtered through a 70 μM cell strainer, and used for experiments.

The experimental procedure was approved by the Latvian Animal Protection Ethical Committee of Food and Veterinary Service (Permit Nr. 93).

### 3.10. BMDMs’ Viability Assay

To determine the cytotoxicity of the SP compound **6e**, the cells were stained with various concentrations of the dye (0, 0.1, 1, 5, 10, and 100 μM). Briefly, 2 × 10^4^ cells were plated in a 96-well plate in 100 μL of the medium. After 24 h, the cells were washed once and a solution of compound **6e** in PBS was added to the cells. The cells were incubated in a humidified 5% CO_2_ incubator at 37 °C for 15–20 min. Then, the dye solution was replaced with medium. The fluorescent imaging was performed using a Leica DM-IL fluorescent microscope with N2.1 filter (excitation: 515–560 nm, emission: 580 nm). After 24 h, the medium was replaced with 10 μL of CCK-8 reagent in 100 μL of fresh medium. The cells were incubated in a humidified 5% CO_2_ incubator at 37 °C for 1–4 h. The absorbance at 450 nm was measured using a microplate spectrophotometer (BioTek Instruments, Winooski, VT, USA). Cell viability was calculated as a percentage of non-treated cell absorbance. IC_50_ values were calculated as the concentration at which 50% of the cells were viable.

### 3.11. Staining of Macrophages with SP Compound ***6e***

For confocal microscopy, BMDMs were stained with 4 μM CellTracker™ CM-DiI dye (Cat. No. C7001; Invitrogen) or 5 μM compound **6e**. Briefly, the cells were washed and then a dye solution in PBS was added to them. The cells were incubated in a humidified 5% CO_2_ incubator at 37 °C for 15–20 min. Then, the cells were stained with WGA Alexa Fluor 488 (Cat. No. W11261; Invitrogen) following the manufacturer’s instructions. Briefly, the cells were incubated with 1 μg/mL WGA solution in PBS at 37 °C. After 10 min, the cells were washed several times, fixed with 3% PFA for 20 min, and stained with DAPI. The samples have been studied under confocal laser scanning microscopy using a Leica TCS SP8 (Leica Microsystems, Wetzlar, Germany, a 63.7 μm pinhole and a 400 Hz scan speed). The fluorescence was detected as follows: a DPSS (561 nm) laser was used for excitation of compound **6e** and CM-Dil, an argon (488 nm) laser was used for WGA, and a diode (405 nm) laser was used for DAPI. For fluorescence detection, HyD was used for compound **6e** and CM-Dil (578 nm–636 nm) and PMT detectors were used for WGA (493 nm–739 nm) and DAPI (417 nm–498 nm), pinhole at 95 µm. The images were analysed using Leica Application Suite X 3.1.5. software (Leica Microsystems, Germany).

### 3.12. Polarisation of Pre-Stained Macrophages to M1 Phenotype

For staining, the cells were seeded onto a 12-well untreated cell culture plate in complete BMDM cultivation medium (RPMI-1640 with 10% FBS and 30% L929-CM containing M-CSF, 2 mM L-glutamine, 1% penicillin/streptomycin) at a concentration of 4 × 10^5^ cells in 1 mL and cultured for 2 days at 37 °C 5% CO_2_. Then, the cell medium was replaced with 1 mL DMEM containing 0.5 µM, 15 µM, or 50 µM compound **6e**. The cells were incubated for 15 min at 37 °C and 5% CO_2_, then the labelling solution was removed, the cells washed with 1.5 mL PBS three times, and 1 mL complete BMDM medium was added. The cells were further used for the functional analysis of pre-stained macrophage polarisation to the M1 phenotype.

For polarisation of macrophages to the pro-inflammatory M1 phenotype, the BMDM cells stained in a 12-well plate were used. Unstained BMDM cells were used as a positive control. The BMDM complete medium from stained and unstained cells was replaced with 1 mL BMDM medium (10% L929 CM containing M-CSF) supplemented with 50 ng/mL recombinant mouse IFNγ (Cat. No. BMS326; eBioscience, San Diego, CA, USA) and 100 ng/mL Pam3SCK4 (Cat. No. tlrl-pms; InvivoGen, Hong Kong, China), and the cells were incubated for 2 days at 37 °C and 5% CO_2_. The BMDM cells cultured in a complete BMDM medium without the IFNγ and Pam3SCK4 activation ligands were incubated in parallel and used as a negative control (M0). After two days of activation, the nitric oxide levels (as a main signal of M1 polarisation) were measured in cell supernatants of activated (M1) and non-activated (M0) cells. The amount of nitric oxide was determined via a nitric oxide assay kit (Cat. No. EMSNO; Invitrogen). Briefly, 50 µL of cell culture media was collected from each well, clarified by centrifugation, and used for NO quantification. The nitrite standards provided by the kit were used for standard curve generation and NO quantification. The optical density was measured at 540 nm using a BioTek µQuant Microplate spectrophotometer (BioTek instruments, USA). The data are presented as the mean value of two independent experiments, and each measurement was performed in triplicate.

### 3.13. BMDM Co-Cultivation with Cancer Cells

For 3D cultivation, the BMDMs were detached from a 12-well plate, resuspended in DMEM at concentration 3 × 10^5^ in 1 mL DMEM, and incubated in the presence of 5 µM compound **6e** for 15 min at 37 °C and 5% CO_2_. Then, the cells were washed three times with PBS, resuspended in 100 µL of complete BMDM medium, and further cultivated in a 3D ultra-low attachment plate together with 4T1/eGFP cancer spheroids that had been prepared as previously described [37]. Briefly, 4T1/eGFP spheroids were generated from 4T1-Fluc-Neo/eGFP-Puro cells (4T1/eGFP; Imanis Life Sciences, Rochester, MN, USA) by seeding 3 000 cells into 96-well black round-bottom ultralow attachment plates (Cat. No. CLS4515, Corning, Life Sciences) according to the manufacturer’s instructions. The next day, the stained macrophages (1.5 × 10^4^ in 100 µL of BMDM medium) were added to the spheroids and the cells cultured for 1–2 days before confocal imaging. Confocal laser scanning microscopy (Leica Microsystems, Germany) was performed as described above: eGFP excitation laser = 488 nm and emission detector = PMT 2 (493–560 nm).

### 3.14. Statistical Analysis

Data statistical analysis was performed using GraphPad Prism 8.0.2. Data are presented as mean ± standard error of the mean (SEM). Statistical analysis was performed using a two-tailed *t*-test.

## 4. Concluding Remarks

In this study, we designed new styrylpyridinium derivatives with varying electron-donating substituents at the phenyl moiety, lengths of alkyl chain at the EWG *N*-alkylpyridinium fragment, and either vinyl or divinyl linkers between them and *bis*-styrylpyridinium derivatives with two styrylpyridinium moieties conjugated through the 1,3-trimethylene unit. A total of nine compounds were synthesised and subsequently evaluated for their spectral, self-assembling, and biological properties.

To reduce phototoxicity and background fluorescence from biomolecules (autofluorescence), a desired fluorescent molecule for bioimaging should be excitable and also emissive in the longer wavelength area, preferentially from the far-red to NIR region (650–950 nm). Currently, NIR-emitting cyanine dyes are widely employed on the market for bioimaging; however, these dyes have disadvantages such as photobleaching, toxicity, and the propensity to produce nonfluorescent aggregates. The new class of fluorescent compounds developed in this study possess relatively high cytotoxicity towards the cancerous cell lines HT-1080 and MH-22A, and at the same time their basal cytotoxicity to the NIH3T3 cell line is evaluated as being from toxic to harmful. The compounds also have intensive fluorescence, especially in lipophylic media. The introduction of a divinyl linker and a powerful electron-donating julolidine moiety into the molecules resulted in intensive fluorescence in the NIR region (maximum at ~760 nm) for compounds **6c**, **e**, and **7b**.

Compound **6e**, comprising a julolidine moiety as electron-donating part, a divinyl linker, and a *N*-hexadecylpyridinium group as the electron-withdrawing part, is proposed as a cell labelling reagent after evaluation using fluorescence microscopy and flow cytometry analysis. Compound **6e** exhibited the remarkable stability and a high stain index even after cell washing, with a stain index of (92 ± 19) before cell washing and (77 ± 15) after cell washing. Furthermore, it was observed that compound **6e** localises within the cell cytoplasm and does not stain the cell nucleus. Based on our findings, it can be inferred that compound **6e** is predominantly taken up by cells through passive transport mechanisms. Amphiphiles, such as SP compound **6e**, exhibit unique properties, allowing them to interact with and stain a diverse range of molecules (both hydrophilic and hydrophobic), making them versatile binding agents. The dye’s ability to remain within the cell further confirms its stability and potential suitability for various experimental applications.

Additionally, staining primary BMDMs with compound **6e** was performed. The staining only slightly affected their viability and functional properties. Importantly, throughout the staining process the BMDMs remained functionally active, as demonstrated by the functional assay results showing no significant impact on gene expression, particularly at low concentrations. This finding underscores the reliability and biocompatibility of SP compound **6e** as a staining agent, making it a valuable tool for cellular studies with promising potential in cell biology research and various biomedical applications.

The development of new fluorescent probes may involve the design of theranostic formulations with multiple functions, including targeted drug delivery and molecular tracking properties.

## Figures and Tables

**Table 1:** Substituents of compounds **6a**–**6g** and **7a**,**b**.

Entry	Compound	Ar	R	n	Ref
1	**6a**	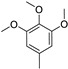	C_12_H_25_	1	
2	**6b**		C_16_H_33_	1	[38]
3	**6c**	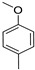	C_16_H_33_	2	
4	**6d**	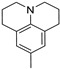	C_12_H_25_	1	
5	**6e**		C_16_H_33_	2	
6	**6f** (**DSP-6**) *	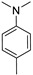	C_6_H_13_	1	[39]
7	**6g** (**DSP-12**) *		C_12_H_25_	1	[39]
8	**7a**	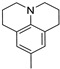	-	1	[40]
9	7b *		-	2	

* Counter ion is tosylate.

**Table 2:** Cytotoxic effect of the compounds (IC_50_, µg/mL) after 72 h treatment of HT-1080 (human connective tissue fibrosarcoma) and MH-22A (mouse hepatocarcinoma) cell lines and basal cytotoxicity (LD_50_) towards the NIH3T3 (mouse fibroblast) cell line.

Entry	Compound	Ar	R	n	IC_50_ (µg/mL)		Basal Cytotoxicity (NIH3T3) LD_50_ mg/kg
					HT-1080	MH-22A	
1	**6a**	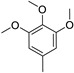	C_12_H_25_	1	0.22 ± 0.06	0.45 ± 0.08	249 ± 3
2	**6b**		C_16_H_33_	1	0.18 ± 0.02	1.08 ± 0.06	91 ± 6
3	**6c**	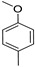	C_16_H_33_	2	0.20 ± 0.04	0.13 ± 0.03	85 ± 7
4	**6d**	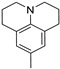	C_12_H_25_	1	1.4 ± 0.3	0.050 ± 0.003	169 ± 18
5	**6e**		C_16_H_33_	2	1.0 ± 0.03	0.40 ± 0.06	477 ± 11
6	**6f** (**DSP-6**) *	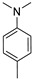	C_6_H_13_	1	0.62 ± 0.06	0.062 ± 0.008	413 ± 25
7	**6g** (**DSP-12**) *		C_12_H_25_	1	0.40 ± 0.1	0.50 ± 0.1	203 ± 32
8	**7a**	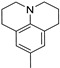	-	1	2.0 ± 0.3	4.0 ± 0.3	1102 ± 20
9	7b *		-	2	6.0 ± 0.2	5.0 ± 0.6	857 ± 13

* The counter ion is tosylate.

**Table 3:** Spectral properties of compounds (fluorescence maximum (nm) in DMSO solution and excitation maximum (nm)) and UV data (extinction coefficient (log ε) and absorption maximum (nm)). The concentrations of compounds **6a**, **b**, **d**, **f**, and **g** were 0.33 mM and of compounds **6c** and **e** were 5 mM for UV measurements. Values of Stokes’ shift (nm) and photoluminescence quantum yields (PLQY) (%).

Entry	Compound	6a	6b	6c	6d	6e	6f	6g	7a	7b
1	fluor. max. λ, nm (excitation. λ, nm)	580 (380)	580 (380)	760 (530)	660 (530)	760 (530)	n	n	660 (530)	770 (530)
2	log ε (abs. max., λ, nm)	5.46 (384)	5.23 (385)	4.26 (408)	5.06 (365) 5.05 (517)	4.02 (520)	5.09 (476)	5.10 (476)	n	n
3	Stokes’ shift, nm	196	195	352	325	240	n	n	n	n
4	PLQY, %	15.1	12.2	0.1	0.5	0.7	1.6	1.4	n	n

n—not measured.

**Table 4:** Values for polydispersity index (PDI) and average diameter (D_av_) of formed nanoparticles; the final concentration of samples was 0.1 mM.

Compound	6a	6c	6d	6e	6g (DSP-12)
D_av_, nm	597 ± 115	212 ± 4	349 ± 4	118 ± 4	207 ± 6
PDI	0.331 ± 0.261	0.218 ± 0.026	0.286 ± 0.030	0.260 ± 0.011	0.254 ± 0.008

## Data Availability

Data is contained within the article.

## Supplementary Materials

The following supporting information can be downloaded at: https://www.mdpi.com/article/10.3390/ph16091245/s1, Scheme S1. Synthesis of 4-methylpyridinium salts; Scheme S2. Synthesis of acrylaldehydes; Scheme S3. Synthesis of compounds **6** and **7**; Table S1. Substituents of compounds **6** and **7**; methods of synthesis; Table S2. Cytotoxicity (IC_50_, µg/mL) on 9 cancer and 3 non-cancerous cell lines and calculated toxicity (LD_50_) of styryl derivatives of **6**; Figure S1. ^1^H NMR spectrum of 1-dodecyl-4-(3,4,5-trimethoxystyryl)pyridin-1-ium bromide (**6b**); Figure S2. ^13^C NMR spectrum of 1-dodecyl-4-(3,4,5-trimethoxystyryl)pyridin-1-ium bromide (**6b**); Figure S3. HRMS data of 1-dodecyl-4-(3,4,5-trimethoxystyryl)pyridin-1-ium bromide (**6b**); Figure S4. ^1^H NMR spectrum of 1-hexadecyl-4-(4-(4-methoxyphenyl)buta-1,3-dien-1-yl)pyridin-1-ium bromide (**6c**); Figure S5. ^13^C NMR spectrum of 1-hexadecyl-4-(4-(4-methoxyphenyl)buta-1,3-dien-1-yl)pyridin-1-ium bromide (**6c**); Figure S6. HRMS data of 1-hexadecyl-4-(4-(4-methoxyphenyl)buta-1,3-dien-1-yl)pyridin-1-ium bromide (**6c**); Figure S7. ^1^H NMR spectrum of 1-dodecyl-4-(2-(2,3,6,7-tetrahydro-1H,5H-pyrido[3,2,1-ij]quinolin-9-yl)vinyl)pyridin-1-ium bromide (**6d**); Figure S8. ^13^C NMR spectrum of 1-dodecyl-4-(2-(2,3,6,7-tetrahydro-1H,5H-pyrido[3,2,1-ij]quinolin-9-yl)vinyl)pyridin-1-ium bromide (**6d**); Figure S9. HRMS data of 1-dodecyl-4-(2-(2,3,6,7-tetrahydro-1H,5H-pyrido[3,2,1-ij]quinolin-9-yl)vinyl)pyridin-1-ium bromide (**6d**); Figure S10. ^1^H NMR spectrum of 1-hexadecyl-4-(4-(2,3,6,7-tetrahydro-1H,5H-pyrido[3,2,1-ij]quinolin-9-yl)buta-1,3-dien-1-yl)pyridin-1-ium bromide (**6e**); Figure S11. ^13^C NMR spectrum of 1-hexadecyl-4-(4-(2,3,6,7-tetrahydro-1H,5H-pyrido[3,2,1-ij]quinolin-9-yl)buta-1,3-dien-1-yl)pyridin-1-ium bromide (**6e**); Figure S12. HRMS data of 1-hexadecyl-4-(4-(2,3,6,7-tetrahydro-1H,5H-pyrido[3,2,1-ij]quinolin-9-yl)buta-1,3-dien-1-yl)pyridin-1-ium bromide (**6e**); Figure S13. ^1^H NMR spectrum of 1,1′-(propane-1,3-diyl)bis(4-(4-(2,3,6,7-tetrahydro-1H,5H-pyrido[3,2,1-ij]quinolin-9-yl)buta-1,3-dien-1-yl)pyridin-1-ium) dibromide (**7b**); Figure S14. ^13^C NMR spectrum of 1,1′-(propane-1,3-diyl)bis(4-(4-(2,3,6,7-tetrahydro-1H,5H-pyrido[3,2,1-ij]quinolin-9-yl)buta-1,3-dien-1-yl)pyridin-1-ium) dibromide (**7b**); Figure S15. HRMS data of 1,1′-(propane-1,3-diyl)bis(4-(4-(2,3,6,7-tetrahydro-1H,5H-pyrido[3,2,1-ij]quinolin-9-yl)buta-1,3-dien-1-yl)pyridin-1-ium) dibromide (**7b**); Figure S16. Three-dimensional excitation emission fluorescent spectral scan of compound **6e**: (**A**) in DMSO (0.5 mM); (**B**) in glycerol (0.01 mM); (**C**) in water (0.5 mM); (**D**) in 0.1 M HCl (0.5 mM); (**E**) in 0.1 M NaOH (0.5 mM) and **DFT calculations**. References [8,38,39,40,75,76,77,78,79,80,81,82,83,84,85] are cited in the supplementary materials.
