# Supplementary material for: Styrylpyridinium Derivatives for Fluorescent Cell Imaging
Source: Pharmaceuticals (Basel). 2023 Sep 4;16(9):1245. doi: 10.3390/ph16091245 (PMC10535741; doi:10.3390/ph16091245)
Supplement: Supplementary file 1 [file pharmaceuticals-16-01245-s001.zip › pharmaceuticals-2480701-supplementary.pdf]

Supplementary material

Styrylpyridinium Derivatives for Fluorescent Cell Imaging

Reinis Putralis [1,2,†], Ksenija Korotkaja [3,†], Martins Kaukulis [1,4], Zhanna Rudevica [3], Juris Jansons [3], Olga Nilova [3], Martins Rucins [1], Laura Krasnova [1], Ilona Domracheva [1], Mara Plotniece [2,4], Karlis Pajuste [1], Arkadij Sobolev [1], Felikss Rumnieks [3], Laura Bekere [1], Anna Zajakina [3], Aiva Plotniece [1,2,*] and Gunars Duburs [1,*]

1 Latvian Institute of Organic Synthesis, LV-1006 Riga, Latvia; reinis.putralis@osi.lv (R.P.); martins.kaukulis@osi.lv (M.K.); rucins@osi.lv (M.R.); chernova@osi.lv (L.K.); ilona@farm.osi.lv (I.D.);
kpajuste@osi.lv (K.P.); arkady@osi.lv (A.S.); laura.bekere.8@gmail.com (L.B.)

2 Department of Pharmaceutical Chemistry, Faculty of Pharmacy, Riga Stradiņš University, LV-1007 Riga, Latvia; mara.plotniece@rsu.lv

3 Latvian Biomedical Research and Study Centre, LV-1067 Riga, Latvia; ksenija.korotkaja@biomed.lu.lv (K.K.); zanna.rudevica@biomed.lu.lv (Z.R.); jansons@biomed.lu.lv (J.J.); nilovaa.olga@gmail.com (O.N.);
felikss.rumnieks@biomed.lu.lv (F.R.); anna@biomed.lu.lv (A.Z.);

4 Faculty of Materials Science and Applied Chemistry, Riga Technical University, LV-1048 Riga, Latvia

* Correspondence: aiva@osi.lv (A.P.); gduburs@osi.lv (G.D.); Tel.: +371-6701-4852(A.P.);

† These authors contributed equally to this work.

1. Synthesis

1.1. *General*

All reagents were purchased from Acros Organics (Geel, Belgium), Alfa Aesar (Lancashire, UK), or Sigma-Aldrich/Merck KGaA (Darmstadt, Germany), and were used without further purification. Thin layer chromatography (TLC) was performed on silica gel 60 F_{254} aluminum sheets 20 cm × 20 cm (Merck KGaA, Darmstadt, Germany) and visualized under UV (254 nm) light and was used to monitor reaction progress. Silica gel of particle size 35–70 µm (Merck KGaA, Darmstadt, Germany) was used for column chromatography for purifications of the compounds. Melting points (mp) were determined on an OptiMelt digital melting point apparatus (Stanford Research Systems, Sunnyvale, CA, USA) and were uncorrected. Proton Nuclear Magnetic Resonance (^{1}H NMR) spectra were recorded with a Bruker Avance Neo (400 MHz) spectrometer and Carbon Nuclear Magnetic Resonance (^{13}C NMR) spectra were recorded with a Bruker Avance Neo (101 MHz) spectrometer (Bruker Biospin Gmbh, Rheinstetten, Germany) or (^{1}H NMR) with Bruker Fourier 300 MHz spectrometer.. The obtained data were used for identification of compound structures. Chemical shifts of the hydrogen and carbon atoms are presented in parts per million (ppm) and referred to as the residual signals of the non-deuterated $CDCl_3$ (δ: 7.26) or partially deuterated DMSO-d_6 (δ: 2.50) solvents for the ^{1}H NMR spectra and $CDCl_3$ (δ: 77.16) or DMSO-d_6 (δ: 39.52) solvents for ^{13}C NMR, respectively. Coupling constants *J* were reported in hertz (Hz). High resolution mass spectra (HRMS) were determined on an Acquity Ultra High Performance Liquid Chromatography (UPLC) H-Class system (Waters, Milford, MA, USA) connected to a Waters Synapt GII Q-ToF operating in the ESI positive ion mode on a Waters Acquity UPLC® BEH C18 column (1.7 µm, 2.1 mm × 50 mm, using gradient elution with acetonitrile (0.1% formic acid) in water (0.1% formic acid).

Citation: Putralis, R.; Korotkaja, K.; Kaukulis, M.; Rudevica, Z.; Jansons, J.; Nilova, O.; Rucins, M.; Krasnova, L.; Domracheva, I.; Plotniece, M.; et al. Styrylpyridinium Derivatives for Fluorescent Cell Imaging. *Pharmaceuticals* **2023**, *16*, x. https://doi.org/10.3390/xxxxx

Academic Editor: Jean-Pierre Bazureau

Received: 15 June 2023
Revised: 25 August 2023
Accepted: 29 August 2023
Published: date

Copyright: © 2023 by the authors. Submitted for possible open access publication under the terms and conditions of the Creative Commons Attribution (CC BY) license (https://creativecommons.org/licenses/by/4.0/).

High Resolution Mass Spectrometry (HRMS) data were used to determine the exact molecular masses of compounds. Flash column chromatography was accomplished using flow chromatography (Armen Instrument—Spot Flash System Interchim, Montluçon, France). UV spectra were recorded on UV-Vis Spectrophotometer (501 UV-Vis CamSpec Spectrophotometer; Spectronic CamSpec Ltd., Leeds, UK). The Dynamic Light Scattering (DLS) measurements of the nanoparticles in the aqueous solution were carried out on a Zetasizer Nano ZSP (Malvern Panalytical Ltd., Malvern, UK) instrument with Malvern Instruments Ltd. Software 8.01.4906. Photoluminescence quantum yields (PLQY) were measured using a standard complementary integrating sphere (the measurement module contains a 150 mm inner diameter integrating sphere for the measurement of fluorescence quantum yields by the absolute method and reflection measurements). The theoretical calculations were performed with Time-dependent density-functional theory (TDDFT) method

1.2. Synthesis of compounds

1.2.1. Synthesis of picolinium derivatives: *N*-alkyl-4-methylpyridin-1-ium bromides **2** and 1,1'-(propane-1,3-diyl)bis(4-methylpyridin-1-ium) dibromide (**3**).

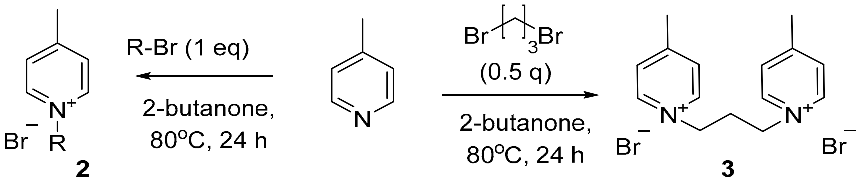

Scheme S1. Synthesis of 4-methylpyridinium salts.

A mixture of 4-methylpyridine (1 eq) and an appropriate alkyl bromide (1 eq) or 1,3-dibromopropane (0.5 eq) in 2-butanone was heated at 80°C for 24 h. When the reaction was completed, it was cooled to 4°C, the precipitate filtered off and recrystalized from acetone.

1-Hexyl-4-methylpyridin-1-ium (**2a**), 1-dodecyl-4-methylpyridin-1-ium (**2b**), 1-hexadecyl-4-methylpyridin-1-ium (**2c**) bromides and 1,1'-(propane-1,3-diyl)bis(4-methylpyridin-1-ium) dibromide (**3**) were obtained with 88%, 64%, 63% and 95% yield, respectively. NMR spectral data for the corresponding salts **2a** [75], **2b** [76],[77], **2c** [76],[78] and **3** [79] were in good agreement with previously published.

1.2.2. Synthesis of acrylaldehydes **5**.

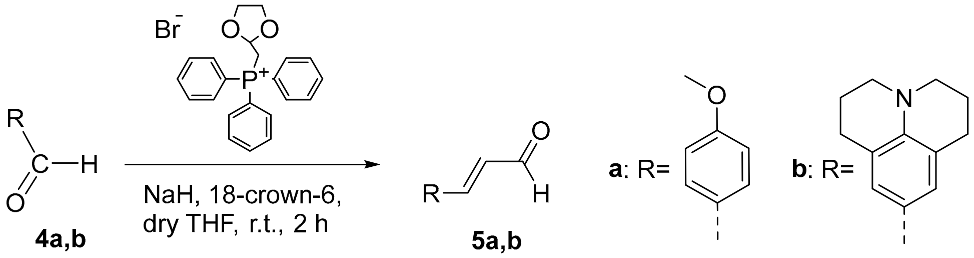

Scheme S2. Synthesis of acrylaldehydes.

NaH (60%, 4 eq) was added to a mixture of the corresponding aldehyde **4** (10 mmol, 1 eq), triphenyl (1,3-dioxolan-2-ylmethyl)phosphonium bromide (5 eq) and 18-crown-6 (0.12 eq) in dry THF (20 mL) and the resulting mixture was stirred at room temperature in a pressure tube under Ar for 2 h. The reaction course was monitored by LC-MS. When all the aldehyde has reacted, the reaction was quenched by the addition of water (20 mL), extracted with diethyl ether (3 × 20 mL). The organic layer was dried over $MgSO_4$, filtered throught celite pad and evaporated to dryness. Afterwards, THF/10 % HCl solution was added to a solid residue and stirred at rt for an additional 0.5 h and again extracted with diethyl ether (3 × 20 mL). The organic

layer was filtered throught celite pad, dried over $MgSO_4$. After solvent evaporation a crude product was purified by flash chromatography using C18 column, eluent - MeCN:water, 1:1 to provide the corresponding aldehydes **5** as solids.

4-Methoxycinnamaldehyde (**5a**) and 3-(2,3,6,7-tetrahydro-1H,5H-pyrido[3,2,1-ij]quinolin-9-yl)acrylaldehyde (**5b**) were obtained with 67% and 73% yield, respectively. NMR spectral data for corresponding aldehydes **5a** [80],[81] and **5b** [82] were in the good agreement with previously published data.

1.2.3. Synthesis of styrylpyridinium **6** and *bis*-styrylpyridinium **7** salts.

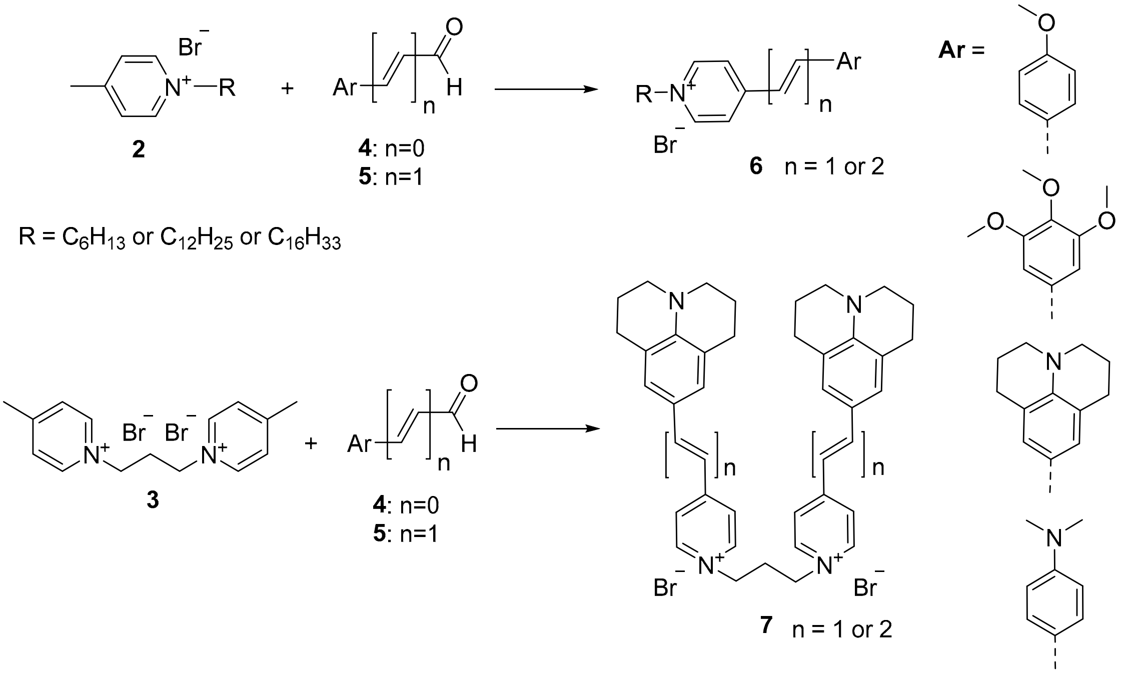

Scheme S3. Synthesis of compounds **6** and **7**. Structures of compounds are depicted in Table S1. Compounds **6f,g** as tosylate.

Table S1. Substituents of synthesized compounds **6** and **7**; methods of synthesis.

Entry	Comp.	Ar	R	n	Method	Ref
1	**6a**	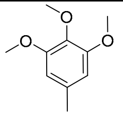	$C_{12}H_{25}$	1	**A**	
2	**6b**		$C_{16}H_{33}$	1	**A**	[38]
3	**6c**	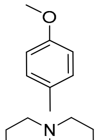	$C_{16}H_{33}$	2	**B**	
4	**6d**	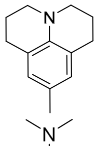	$C_{12}H_{25}$	1	**A**	
5	**6e**		$C_{16}H_{33}$	2	**B**	
6	**6f (DSP-6)**	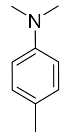	C_6H_{13}	1	**A**	[75],[39]
7	**6g (DSP-12)**		$C_{12}H_{25}$	1	**A**	[39]
8	**7a**	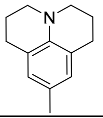	-	1	**A**	[40]
9	**7b**		-	2	**B**	

Method A. Compounds **6a,b,d,f,g** and **7a** were synthesized in analogy with the procedure described by Vaitkiene et al. [8]. Briefly, the mixture of the appropriate *N*-alkyl-4-methylpyridinium bromide **2** (1 eq), aldehyde **4** or **5** (0.25 mmol, 1.3 eq), and piperidine (3 drops) in ethanol (20 mL) was refluxed for 24 h. Reaction course was monitored by TLC and LC-MS. The solvent was evaporated to dryness. The residue was triturated with diethyl ether and cooled to +4°C and sonicated by bath-type sonicator (Cole Parmer Ultrasonic Cleaner 8891CPX (Vernon Hills, IL, USA)). The solvent was decanted, and the procedure was repeated three times. In final, the precipitate was filtered and recrystallized from 2-butanone.

1-Dodecyl-4-(3,4,5-trimethoxystyryl)pyridin-1-ium bromide (**6a**)

Yield 35%. Yellow solid, m.p.146-147°C. ^{1}H NMR (300 MHz, DMSO-d$_6$) δ 8.95 (d, *J* = 6.9 Hz, 2H), 8.19 (d, *J* = 6.9 Hz, 2H), 7.98 (d, *J* = 16.3 Hz, 1H), 7.52 (d, *J* = 16.3 Hz, 1H), 7.10 (s, 2H), 4.48 (t, J = 7.4 Hz, 2H), 3.86 (s, 6H), 3.73 (s, 3H), 1.97 – 1.84 (m, 2H), 1.36 – 1.14 (m, 18H), 0.90 – 0.79 (m, 3H) ppm. ^{13}C NMR (101 MHz, DMSO-d$_6$) δ 153.2, 152.9, 144.2, 141.1, 139.6, 130.7, 123.5, 122.5, 105.7, 60.2, 59.7, 56.0, 31.3, 30.5, 29.0, 28.9, 28.8, 28.7, 28.4, 25.4, 22.1, 13.9 ppm. HRMS (TOF MS ES+): Calculated $[C_{28}H_{42}NO_3 + H]^+$ 440.3165; found: 440.3167.

1-Dodecyl-4-(2-(2,3,6,7-tetrahydro-1H,5H-pyrido[3,2,1-ij]quinolin-9-yl)vinyl)pyridin-1-ium bromide (**6d**)

After sonication crude product was purified by flash chromatography, eluent EtOAc:PE (70:30 till 50:50), after then recrystalized from 2-butanone. Yield 15%. Dark red solid, m.p .113-114°C.

^{1}H NMR (300 MHz, DMSO-d$_6$) δ 8.69 (d, *J* = 6.8 Hz, 2H), 7.97 (d, *J* = 6.8 Hz, 2H), 7.79 (d, *J* = 16.0 Hz, 1H), 7.15 (s, 2H), 7.05 (d, *J* = 16.0 Hz, 1H), 4.37 (t, *J* = 7.3 Hz, 2H), 3.26 (t, *J* = 5.8 Hz, 4H), 2.70 (t, *J* = 6.2 Hz, 4H), 1.93 – 1.81 (m, 6H), 1.30 – 1.19 (m, 18H), 0.87 – 0.81 (m, 3H) ppm. ^{13}C NMR (101 MHz, DMSO-d$_6$) δ 153.7, 145.2, 143.2, 142.6, 127.9, 121.9, 121.4, 120.7, 115.8, 58.9, 49.3, 31.3, 30.4, 29.0, 28.9, 28.8, 28.7, 28.4, 27.1, 25.4, 22.1, 20.9, 14.0 ppm. HRMS (TOF MS ES+): Calculated $[C_{31}H_{45}N_2 + H]^+$ 445.3583; found: 445.3588.

4-(3,4,5-Trimethoxystyryl)-1-hexadecylpyridin-1-ium bromide (**6b**), 4-(4-(dimethylamino)styryl)-1-dodecylpyridin-1-ium bromide (DSP-12, **6g**), 4-(4-(dimethylamino)styryl)-1-hexylpyridin-1-ium (DSP-6, **6f**) and were obtained with 49%, 55% and 61%yield, respectively. NMR spectral data for corresponding salts **6g** [39], **6f** [75],[39], and **6b** [38] were in good agreement with previously published data.

Also 1,1'-(propane-1,3-diyl)bis(4-(-2-(2,3,6,7-tetrahydro-1H,5H-pyrido[3,2,1-ij]quinolin-9-yl)vinyl)-pyridin-1-ium) dibromide (2-2, **7a**) was prepared by the same method used for compounds **6.** Beginning with 1,1'-(propane-1,3-diyl)bis(4-methylpyridin-1-ium) dibromide (**3**) (1 eq) and 2,3,6,7-tetrahydro-1H,5H-pyrido[3,2,1-ij]quinoline-9-carbaldehyde (**4b**) (2 eq) the desired compound was obtained with 44% yield. NMR spectral data for **7a** [38],[83] were in good agreement with previously published data.

Method B. Derivatives with divinyl linker, compounds **6c,e** and **7b** were synthesized by a modified procedure proposed by Coe et al [84]. A mixture of the appropriate N-alkyl-4-methylpyridinium bromide **2** (1 eq) or 1,1'-(propane-1,3-diyl)bis(4-methylpyridin-1-ium) dibromide (**3**) (1 eq), aldehyde **4** or **5** (2.4 eq) was charged in a pressure tube under Ar, then dry MeOH (3 mL) and pyrrolidine (12.2 eq) was added and after which the reaction mixture was stirred at rt for 2 h. The reaction course was monitored by TLC. The reaction was quenched by the addition of diethyl ether (100 mL) and sonicated in bath-type sonicator (Cole Parmer Ultrasonic Cleaner 8891CPX (Vernon Hills, IL, USA)) for 2 min. Sonicated suspension was centrifuged in Bechman coulter optima™ L-80 XP ultracentrifuge (Beckman Coulter Inc, Brea, CA, United States) at 5000 × *g* for 5 min and the supernatant was discarded. The precipitate was then purified by dissolution-precipitation procedure from MeOH with Et_2O (~1:10) The dissolution-precipitation procedure was repeated twice, after which the precipitate was dried in vacuum.

1-Hexadecyl-4-(4-(4-methoxyphenyl)buta-1,3-dien-1-yl)pyridin-1-ium bromide (**6c**)
Yield: 47%, dark orange solid, m.p.154-155°C. ^{1}H NMR (300 MHz, $CDCl_3$) δ 9.06 (d, *J* = 6.8 Hz, 2H), 7.89 (d, *J* = 6.8 Hz, 2H), 7.53 (dd, *J* = 15.3, 10.4 Hz, 1H), 7.45 (d, *J* = 8.8 Hz, 2H), 7.02 (d, *J* = 15.3 Hz, 1H), 6.90 (d, *J* = 8.8 Hz, 2H), 6.87 (dd, *J* = 15.3, 10.4 Hz, 1H), 6.60 (d, *J* = 15.3 Hz, 1H), 4.76 (t, *J* = 7.3 Hz, 2H), 3.84 (s, 3H), 2.03 – 1.92 (m, 2H), 1.32 – 1.15 (m, 26H), 0.90 – 0.83 (m, 3H) ppm. ^{13}C NMR (101 MHz, $CDCl_3$) δ 161.3, 153.7, 144.0, 143.5, 142.8, 129.4, 128.7, 124.9, 124.3, 123.5, 114.6, 77.4, 61.0, 55.6, 32.1, 31.9, 29.8, 29.7, 29.6, 29.5, 29.4, 29.3, 29.2, 26.3, 22.8, 14.3 ppm. HRMS (TOF MS ES+): Calculated [$C_{32}H_{48}NO$ + H]+ 462.3736; found: 462.3739.

1-Hexadecyl-4-(4-(2,3,6,7-tetrahydro-1H,5H-pyrido[3,2,1-ij]quinolin-9-yl)buta-1,3-dien-1-yl)pyridin-1-ium bromide (**6e**)
Yield: 53%, dark violet solid, m.p.145-146°C. ^{1}H NMR (300 MHz, $CDCl_3$) δ 8.88 (d, *J* = 6.5 Hz, 2H), 7.73 (d, *J* = 6.5 Hz, 2H), 7.48 (dd, *J* = 15.2, 10.7 Hz, 1H), 6.96 (s, 2H), 6.89 (d, *J* = 15.2 Hz, 1H), 6.75 (dd, *J* = 15.0, 10.7 Hz, 1H), 6.43 (d, *J* = 15.0 Hz, 1H), 4.66 (t, *J* = 7.4 Hz, 2H), 3.34 – 3.19 (m, 4H), 2.78 – 2.68 (m, 4H), 2.00 – 1.92 (m, 6H), 1.25 – 1.21 (m, 26H), 0.89 – 0.85 (m, 3H) ppm. ^{13}C NMR (101 MHz, $CDCl_3$) δ 153.8, 144.9, 144.8, 144.7, 143.4, 127.4, 122.8, 122.6, 121.6, 121.4, 121.3, 77.4, 60.4, 50.1, 32.0, 31.7, 29.8, 29.7, 29.6, 29.5, 29.4, 29.3, 29.2, 27.8, 26.2, 22.8, 21.6, 14.2 ppm. HRMS (TOF MS ES+): Calculated [$C_{37}H_{55}N_2$ + H]$^+$ 527.4365; found: 527.4362.

1,1'-(Propane-1,3-diyl)bis(4-(4-(2,3,6,7-tetrahydro-1H,5H-pyrido[3,2,1-ij]quinolin-9-yl)buta-1,3-dien-1-yl)pyridin-1-ium) dibromide (**7b**)
Yield: 18%, dark violet solid, m.p.142-143°C. ^{1}H NMR (400 MHz, $CDCl_3$) δ 9.22 – 9.16 (m, 4H), 7.59 (d, *J* = 6.0 Hz, 4H), 7.40 (dd, *J* = 15.0, 10.8 Hz, 2H), 6.94 (s, 4H), 6.86 (d, *J* = 15.0 Hz, 2H), 6.79 – 6.68 (m, 2H), 6.39 (d, *J* = 15.0 Hz, 2H), 4.96 – 4.88 (m, 4H), 3.26 – 3.19 (m, 8H), 2.75 – 2.68 (m, 8H), 1.99 – 1.88 (m, 10H) ppm. ^{13}C NMR (101 MHz, $CDCl_3$) δ 154.0, 145.0, 144.9, 144.6, 143.9, 127.5, 122.8, 122.4, 121.5, 121.4, 121.3, 56.2, 50.1, 34.5, 31.0, 27.8, 21.6 ppm. HRMS (TOF MS ES+): Calculated [$C_{45}H_{50}N_4$]$^+$/2 323.2012; found: 323.2026.

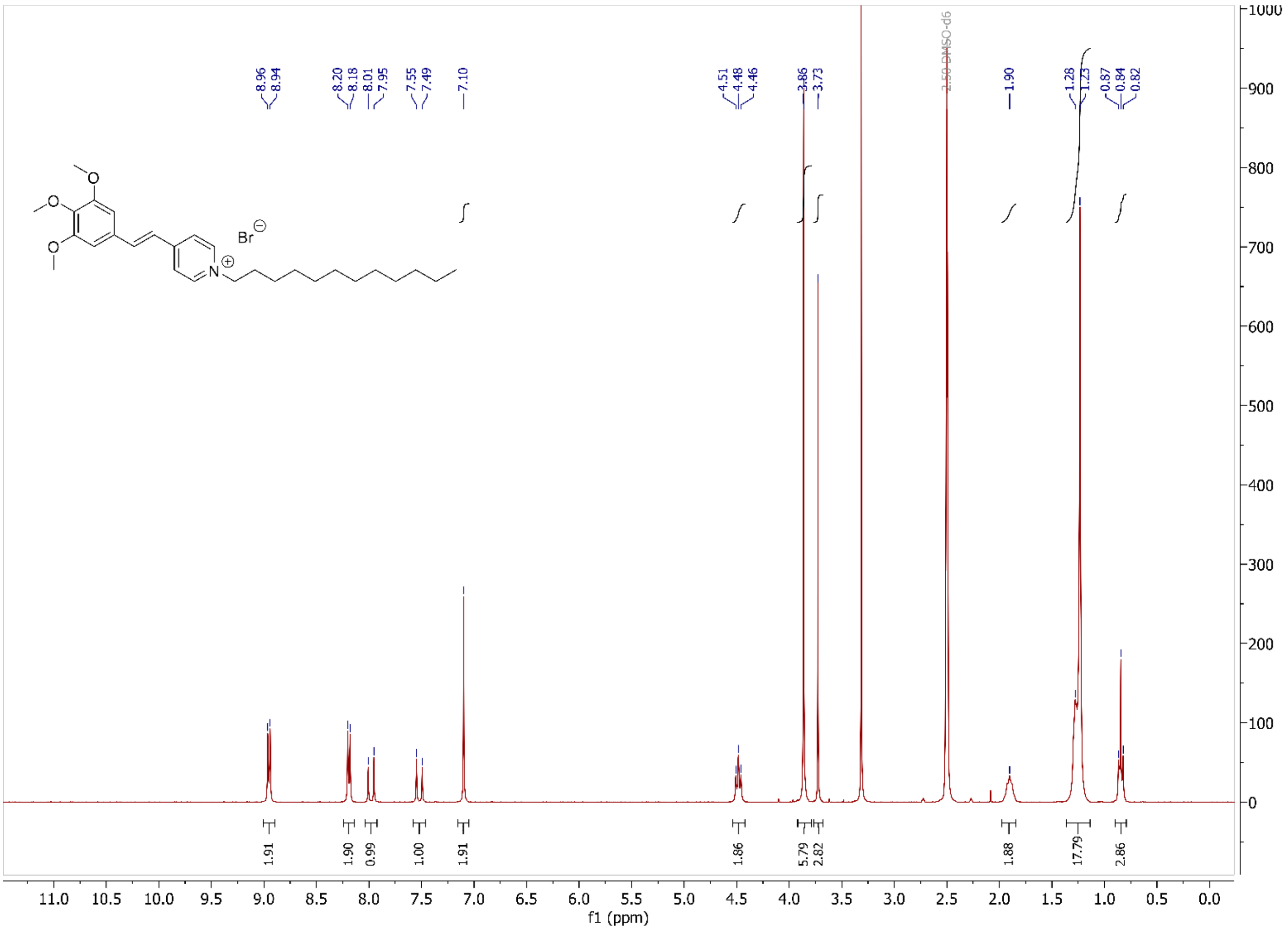

Figure S1. ^1H NMR spectrum of 1-dodecyl-4-(3,4,5-trimethoxystyryl)pyridin-1-ium bromide (**6b**)

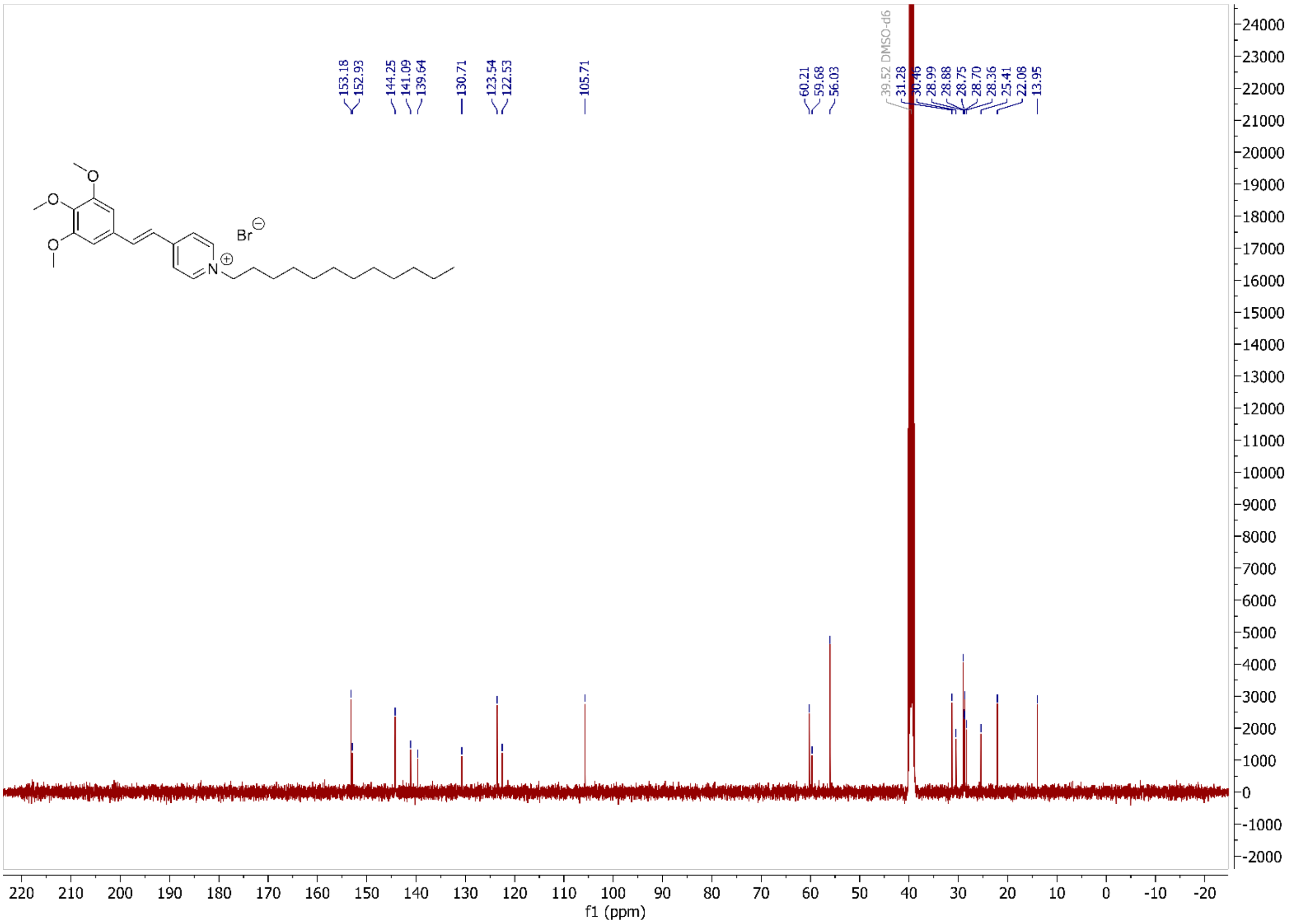

Figure S2. ^{13}C NMR spectrum of 1-dodecyl-4-(3,4,5-trimethoxystyryl)pyridin-1-ium bromide (**6b**)

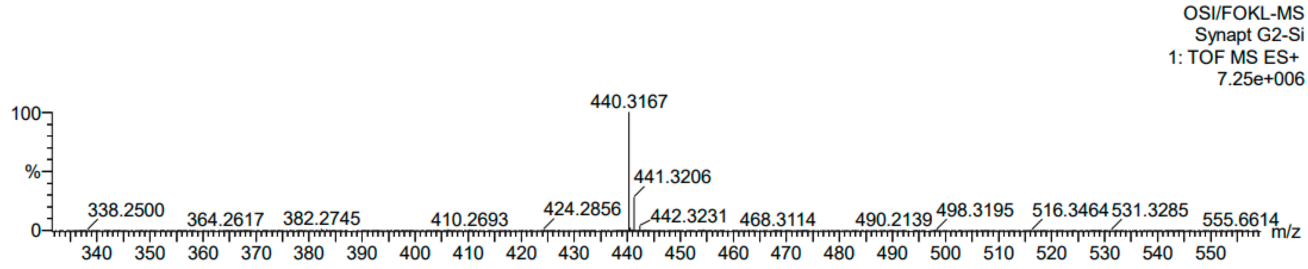

Minimum:				-0.5				
Maximum:		10.0	5.0	200.0				
Mass	Calc. Mass	mDa	PPM	DBE	i-FIT	Norm	Conf(%)	Formula
440.3167	440.3165	0.2	0.5	8.5	917.4	n/a	n/a	C28 H42 N O3

Figure S3. HRMS data of 1-dodecyl-4-(3,4,5-trimethoxystyryl)pyridin-1-ium bromide (**6b**)

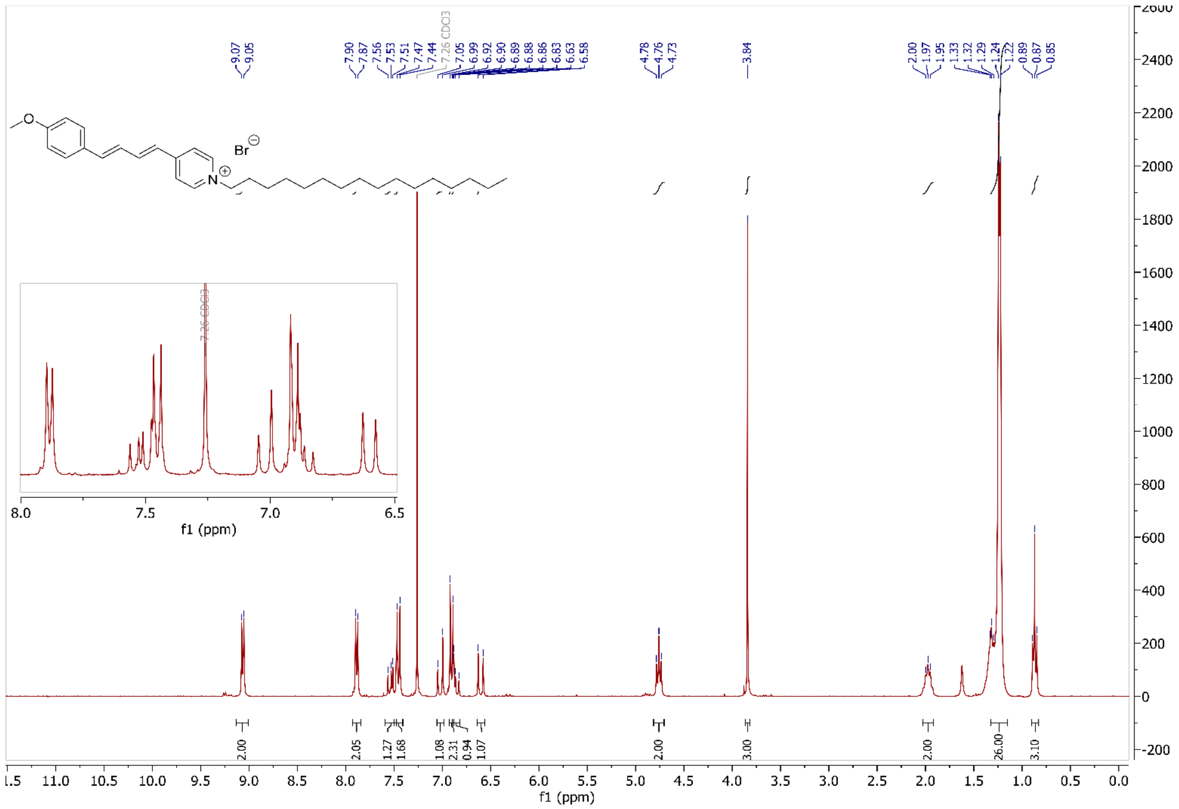

Figure S4. ^{1}H NMR spectrum of 1-hexadecyl-4-(4-(4-methoxyphenyl)buta-1,3-dien-1-yl)pyridin-1-ium bromide (**6c**)

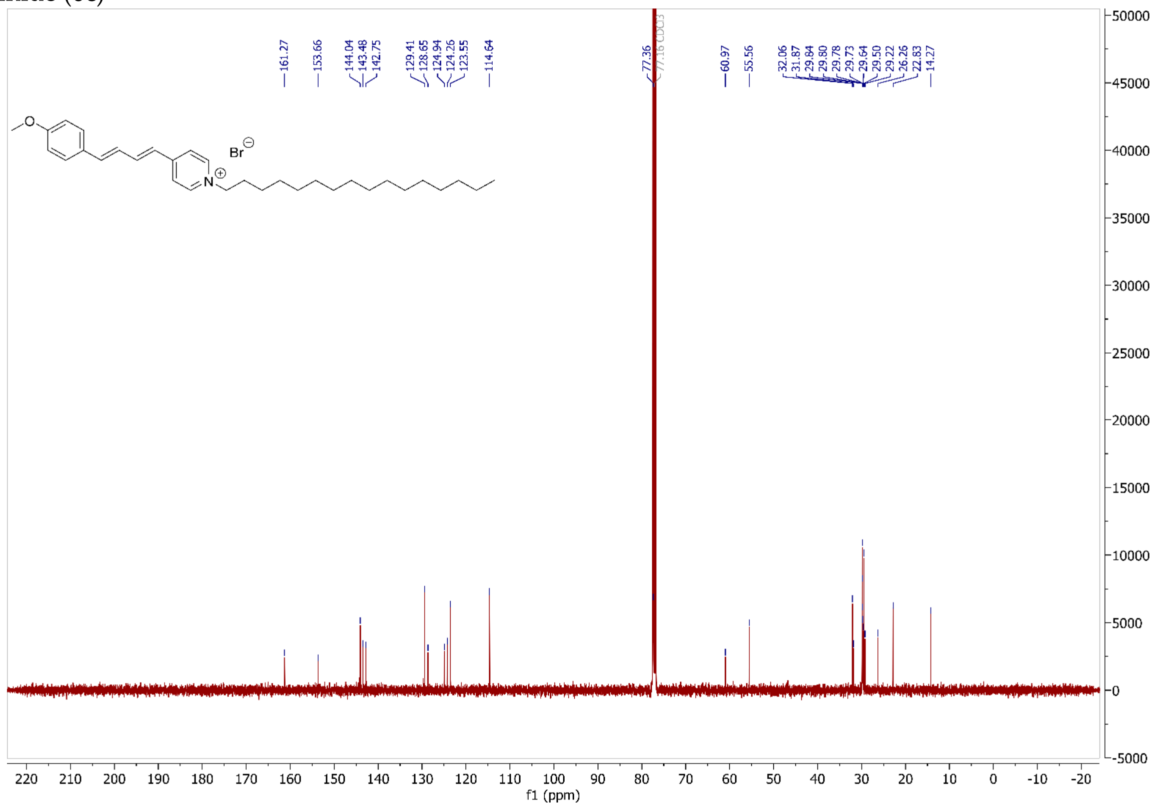

Figure S5. ^{13}C NMR spectrum of 1-hexadecyl-4-(4-(4-methoxyphenyl)buta-1,3-dien-1-yl)pyridin-1-ium bromide (**6c**)

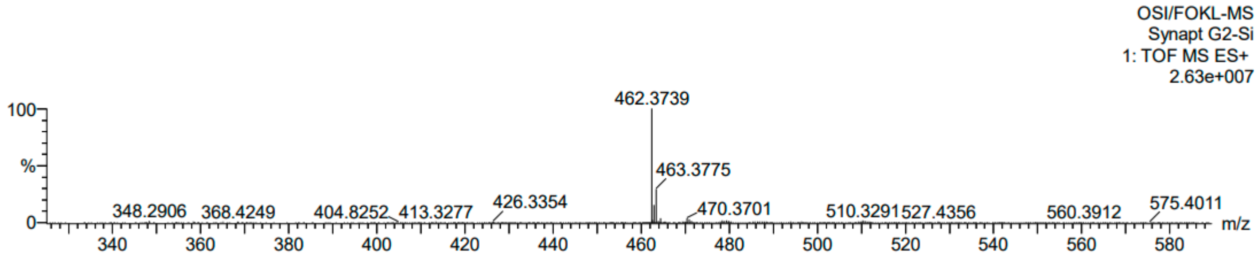

Minimum:				-0.5				
Maximum:		10.0	5.0	200.0				
Mass	Calc. Mass	mDa	PPM	DBE	i-FIT	Norm	Conf(%)	Formula
462.3739	462.3736	0.3	0.6	9.5	976.1	n/a	n/a	C32 H48 N O

Figure S6. HRMS data of 1-hexadecyl-4-(4-(4-methoxyphenyl)buta-1,3-dien-1-yl)pyridin-1-ium bromide (**6c**)

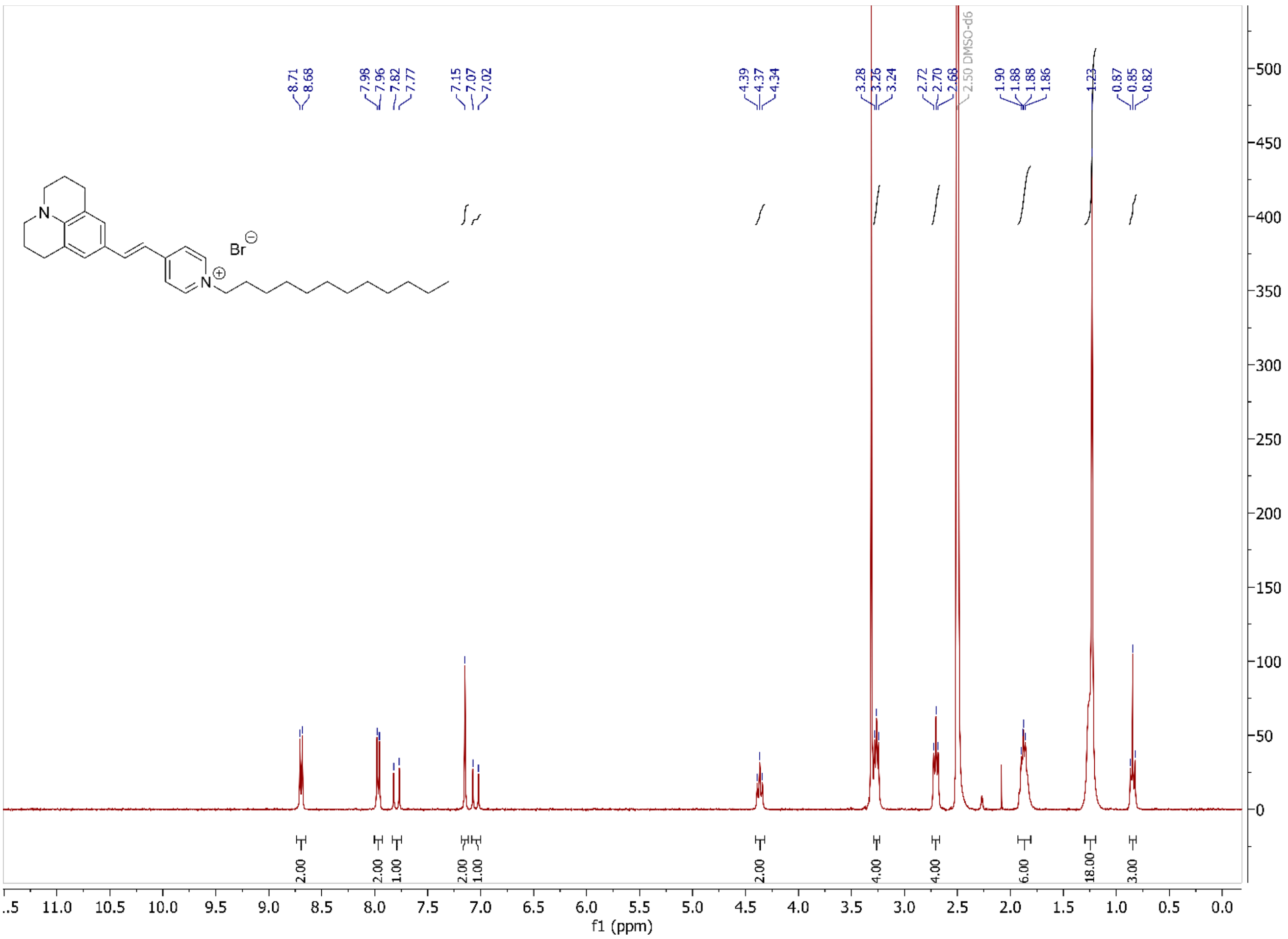

Figure S7. ^{1}H NMR spectrum of 1-dodecyl-4-(2-(2,3,6,7-tetrahydro-1H,5H-pyrido[3,2,1-ij]quinolin-9-yl)vinyl)pyridin-1-ium bromide (**6d**)

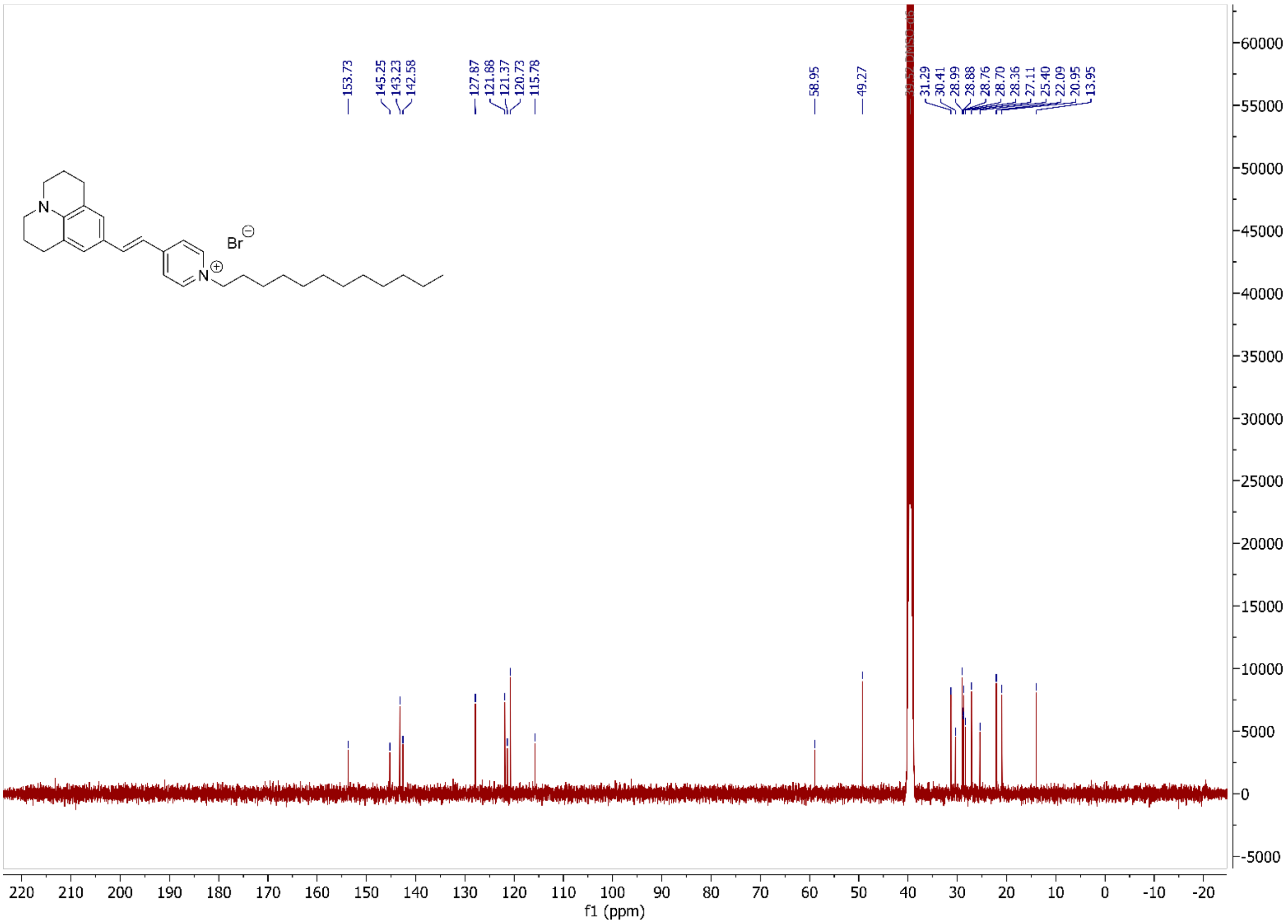

Figure S8. ^{13}C NMR spectrum of 1-dodecyl-4-(2-(2,3,6,7-tetrahydro-1H,5H-pyrido[3,2,1-ij]quinolin-9-yl)vinyl)pyridin-1-ium bromide (**6d**)

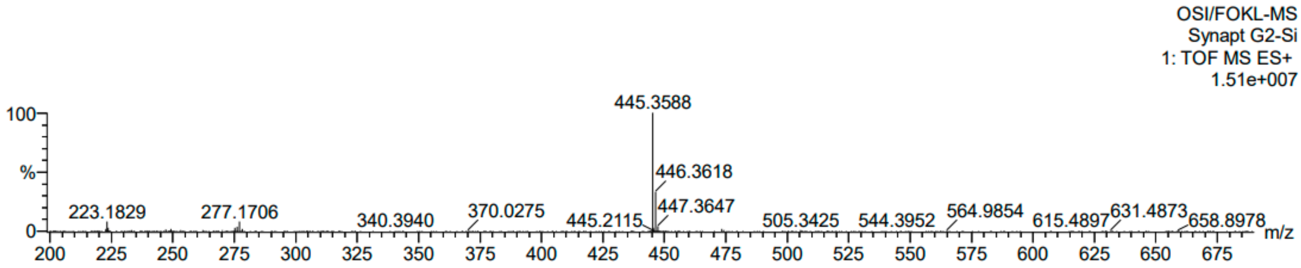

Minimum:				-0.5				
Maximum:		10.0	5.0	200.0				
Mass	Calc. Mass	mDa	PPM	DBE	i-FIT	Norm	Conf(%)	Formula
445.3588	445.3583	0.5	1.1	10.5	638.3	n/a	n/a	C31 H45 N2

Figure S9. HRMS data of 1-dodecyl-4-(2-(2,3,6,7-tetrahydro-1H,5H-pyrido[3,2,1-ij]quinolin-9-yl)vinyl)pyridin-1-ium bromide (**6d**)

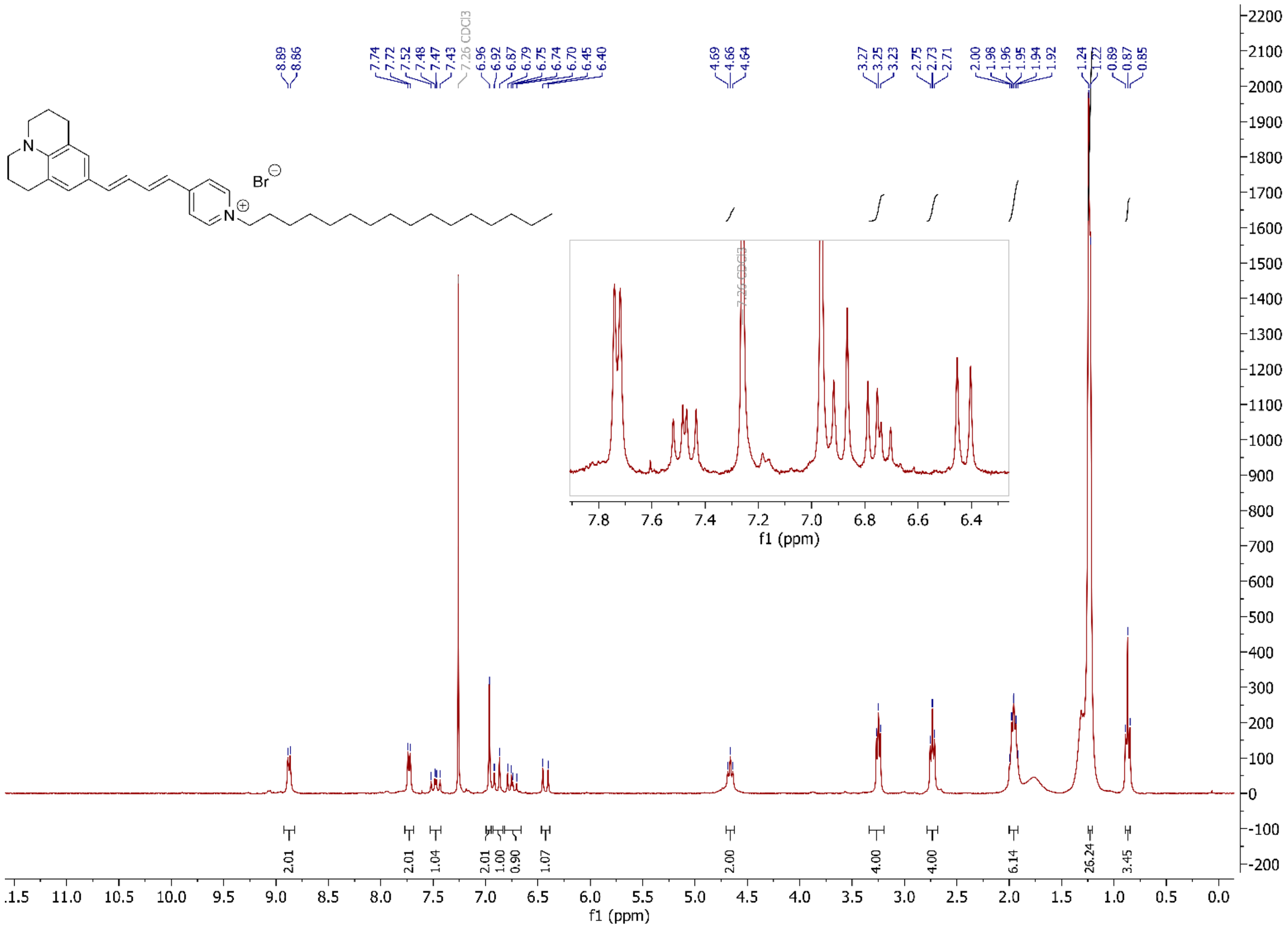

Figure S10. ^{1}H NMR spectrum of 1-hexadecyl-4-(4-(2,3,6,7-tetrahydro-1H,5H-pyrido[3,2,1-ij]quinolin-9-yl)buta-1,3-dien-1-yl)pyridin-1-ium bromide (**6e**)

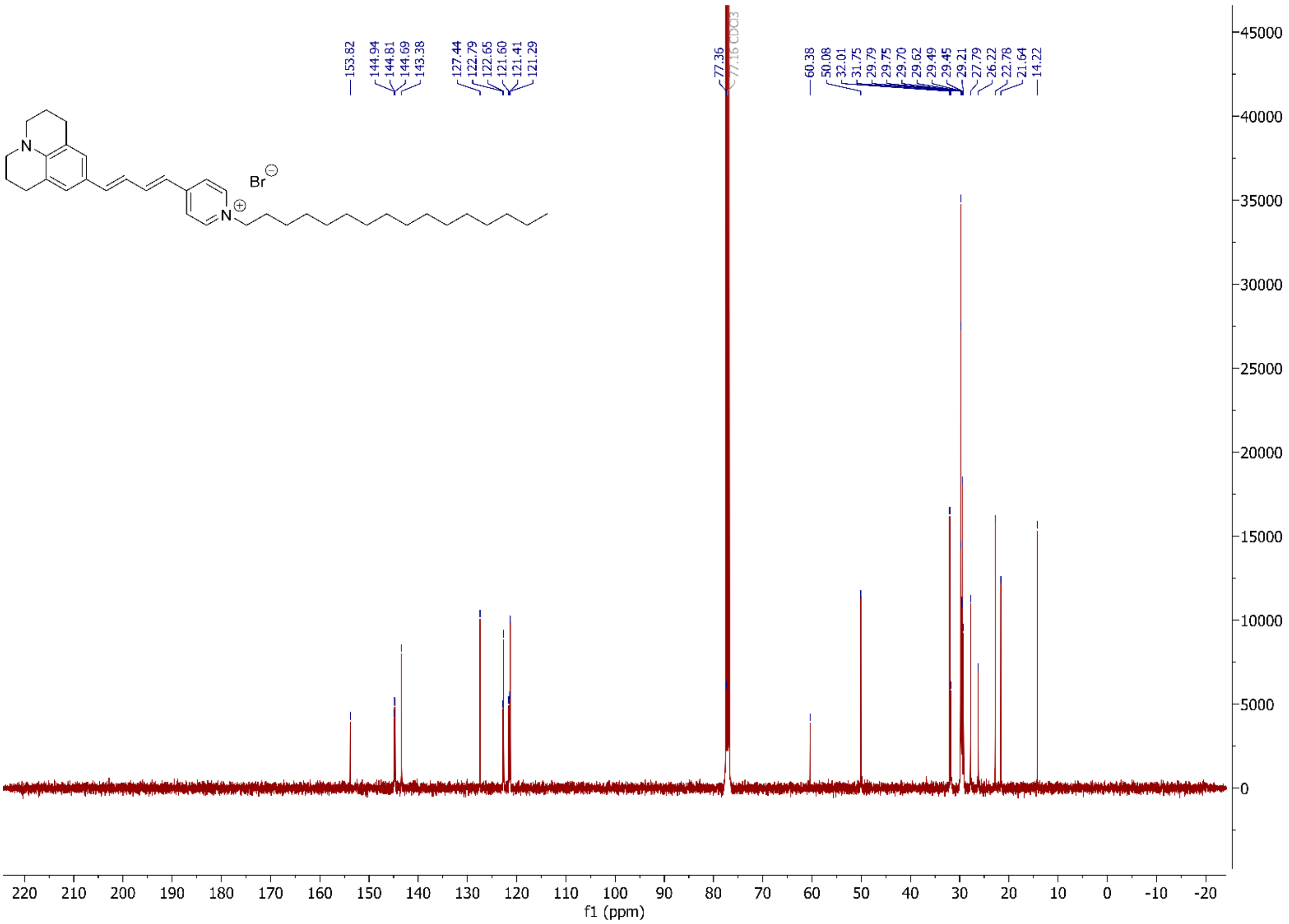

Figure S11. ^{13}C NMR spectrum of 1-hexadecyl-4-(4-(2,3,6,7-tetrahydro-1H,5H-pyrido[3,2,1-ij]quinolin-9-yl)buta-1,3-dien-1-yl)pyridin-1-ium bromide (**6e**)

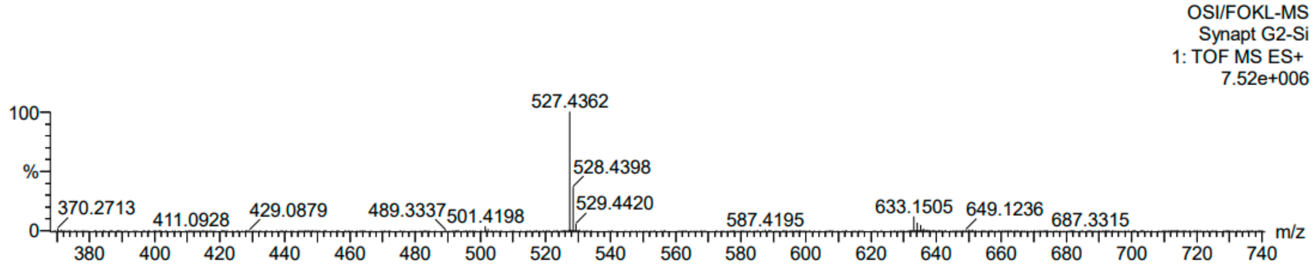

Minimum:				-0.5					
Maximum:		10.0	5.0	200.0					
Mass	Calc. Mass	mDa	PPM	DBE	i-FIT	Norm	Conf(%)	Formula	
527.4362	527.4365	-0.3	-0.6	11.5	699.6	n/a	n/a	C37 H55 N2	

Figure S12. HRMS data 1-hexadecyl-4-(4-(2,3,6,7-tetrahydro-1H,5H-pyrido[3,2,1-ij]quinolin-9-yl)buta-1,3-dien-1-yl)pyridin-1-ium bromide (**6e**)

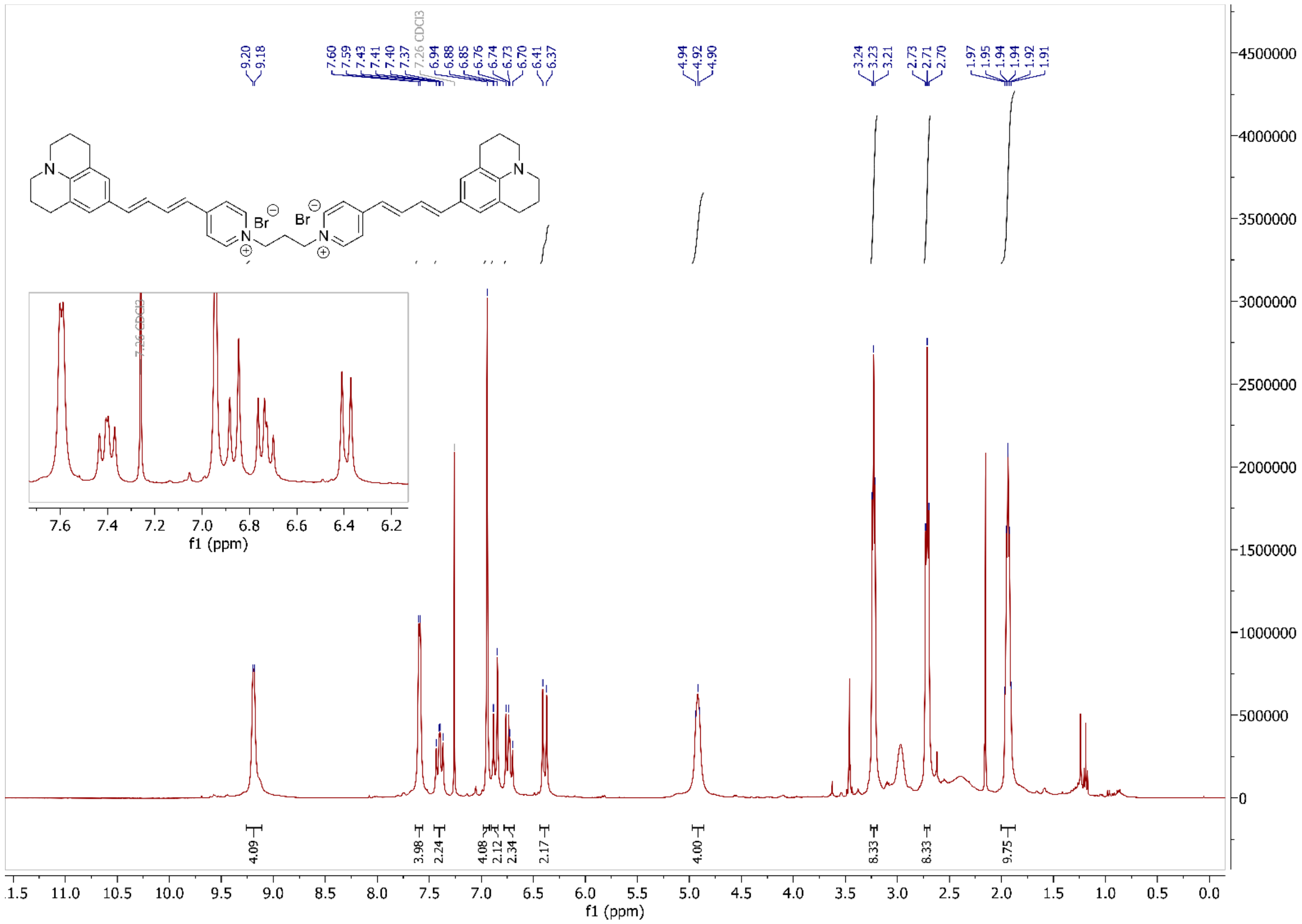

Figure S13. ^{1}H NMR spectrum of 1,1'-(propane-1,3-diyl)bis(4-(4-(2,3,6,7-tetrahydro-1H,5H-pyrido[3,2,1-ij]quinolin-9-yl)buta-1,3-dien-1-yl)pyridin-1-ium) dibromide (**7b**)

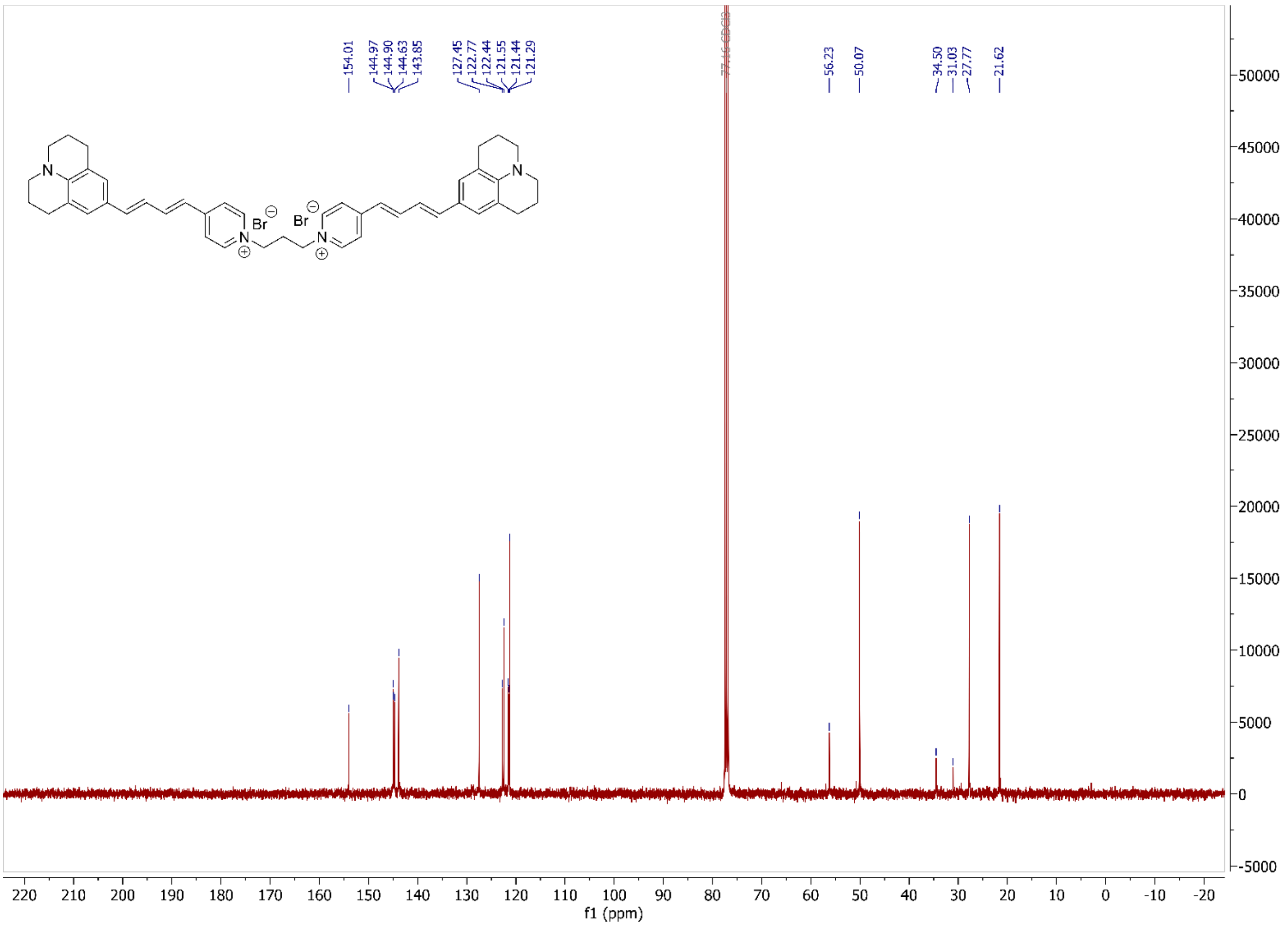

Figure S14. ^{13}C NMR spectrum of 1,1'-(propane-1,3-diyl)bis(4-(4-(2,3,6,7-tetrahydro-1H,5H-pyrido[3,2,1-ij]quinolin-9-yl)buta-1,3-dien-1-yl)pyridin-1-ium) dibromide (**7b**)

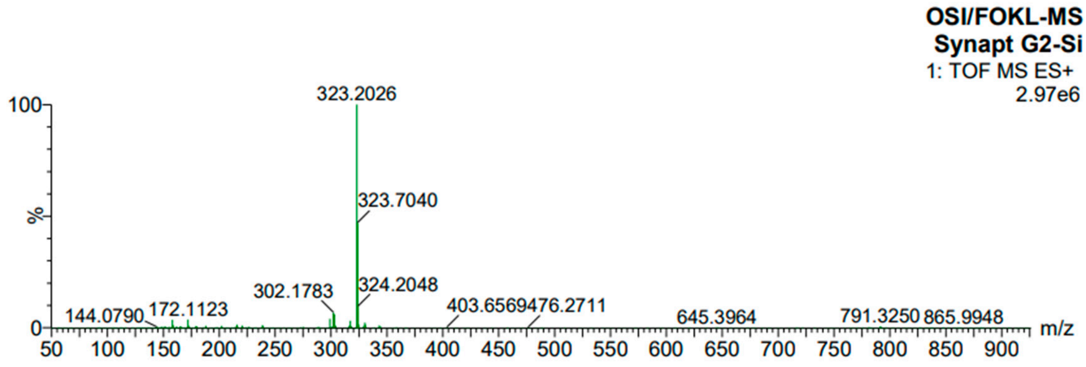

$[C_{45}H_{50}N_4]^{2+}$ (teor.) = 323.2012
$[C_{45}H_{50}N_4]^{2+}$ (found.) = 323.2026

Figure S15. HRMS data of 1,1'-(propane-1,3-diyl)bis(4-(4-(2,3,6,7-tetrahydro-1H,5H-pyrido[3,2,1-ij]quinolin-9-yl)buta-1,3-dien-1-yl)pyridin-1-ium) dibromide (**7b**)

2. Cytotoxicity.

The HT-1080 (human connective tissue fibrosarcoma, ATCC® CCL-121™), MH-22A (mouse hepatocarcinoma, ECACC Cat.No. 96121721), A-549 (human lung carcinoma, ATCC® CCL-185™), NIH 3T3 (mouse Swiss Albino embryo fibroblast, ECACC Cat.No. 93061524), Hek293 (human embryonic kidney, ATCC® CRL-1573™), BHK-21 (hamster Syrian kidney, ATCC® CCL-10™), A-431 (human epidermoid carcinoma, ATCC® CRL-1555™), Saos-2 (human osteosarcoma, ATCC® HTB-85™) HeLa (human epitheloid cervix carcinoma, ATCC® CCL-2™), U937 (human myeloid leukaemia, ATCC® CRL-1593.2™), C6 (rat glioma, ATCC® CCL-107™), B16-F10 (murine melanoma, ATCC® CRL-6475™) cell lines were obtained from American Type Culture Collection (ATCC) and European Collection of Authenticated Cell Cultures (ECACC).

Table S2. Cytotoxicity (IC_{50}, μg/mL) on 9 cancer and 3 non-cancerous cell lines and calculated toxicity (LD_{50}) of styryl derivatives **6.**

**entry**	**comp**	**Cytotoxicity IC_{50}, μg/mL**										**Basal cytotoxicity (NIH3T3) LD_{50}**	
		**"Non-cancerous"**			**Cancer cell line**								
		**BHK-21**	**NIH3T3**	**Hek-293**	**A-431**	**A-549**	**Saos-2**	**HeLa**	**U937**	**C6**	**B16-F10**	**mM/kg**	**mg/kg**
**1**	**6a**	1.01 ±0.10	1.23 ±0.08	0.22 ±0.04	0.87 ±0.05	0.87 ±0.06	0.37 ±0.06	0.59 ±0.02	1.01 ±0.08	0.86 ±0.05	0.80 ±0.08	0.50 ±0.01	249±3
**2**	**6b**	1.11 ±0.05	1.02 ±0.02	0.38 ±0.07	1.10 ±0.08	0.98 ±0.05	0.60 ±0.03	0.82 ±0.04	1.39 ±0.05	1.23 ±0.03	0.63 ±0.09	0.15 ±0.01	91±6
**3**	**6c**	0.35 ±0.10	0.24 ±0.03	0.41 ±0.06	0.58 ±0.07	0.21 ±0.03	0.44 ±0.09	0.33 ±0.08	0.69 ±0.07	0.60 ±0.05	0.49 ±0.01	0.16 ±0.01	85±7
**4**	**6d**	0.082 ±0.005	0.056 ±0.002	0.012 ±0.002	0.098 ±0.03	0.055 ±0.003	0.79 ±0.04	0.055 ±0.008	0.044 ±0.005	0.129 ±0.011	0.113 ±0.008	0.30 ±0.03	169±18
**5**	**6e**	2.70 ±1.00	3.20 ±0.20	1.50 ±0.40	1.90 ±0.04	3.0 ±0.10	3.60 ±0.30	1.30 ±0.30	2.40 ±0.30	3.40 ±0.60	2.90 ±0.05	0.80 ±0.02	477±11
**6**	**6f**	0.48 ±0.01	0.60 ±0.03	0.059 ±0.004	0.56 ±0.10	0.19 ±0.02	1.54 ±0.08	0.23 ±0.02	0.13 ±0.03	0.89 ±0.04	1.80 ±0.05	0.90 ±0.09	413±25

3. Fluorescence

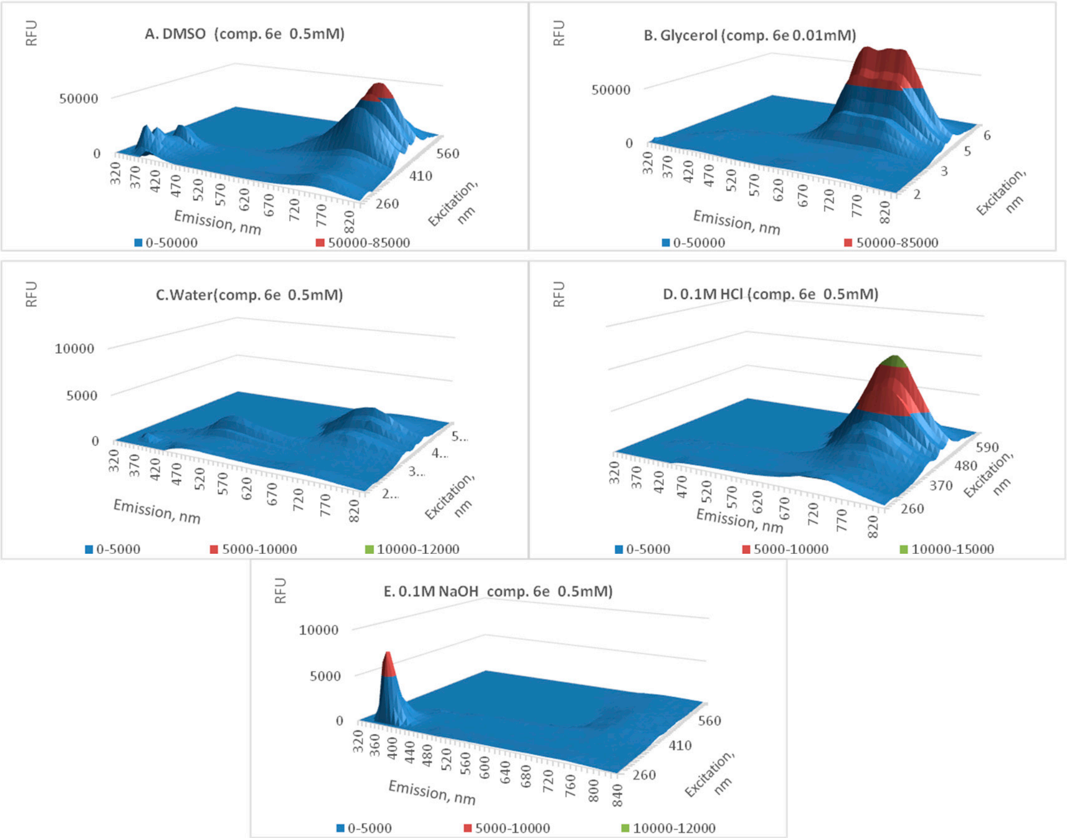

Figure S16. 3D excitation emission fluorescent spectral scan of comp. **6e**: A: in DMSO (0.5 mM); **B:** in glycerol (0.01 mM); **C:** in water (0.5 mM); **D:** in 0.1 M HCl (0.5 mM); E: in 0.1 M NaOH (0.5 mM).

4. DFT calculations

All calculations were performed using Gaussian 09 software package [85]. In order to simplify calculations methyl pyridinium salt was used as a model substrate. First, the optimization of the stationary points was performed without any restrictions using density-functional theory method b3lyp/6-31+g(d). The stationary point was verified to be real minima (zero imaginary frequency) by performing frequency calculations at the same level of theory. Second, time-dependent density-functional theory (TDDFT) calculations were performed on the optimized geometry using b3lyp/6-311++g(2df,p) method to determine absorption spectra. Third, geometry of the exited state S1 was optimized using b3lyp/6-31+g(d). Finally, TDDFT calculations using b3lyp/6-311++g(2df,p) method was performed on the S1 geometry to obtain emission spectra.

4.1. Optimized geometries

4.1.1. Ground state geometry

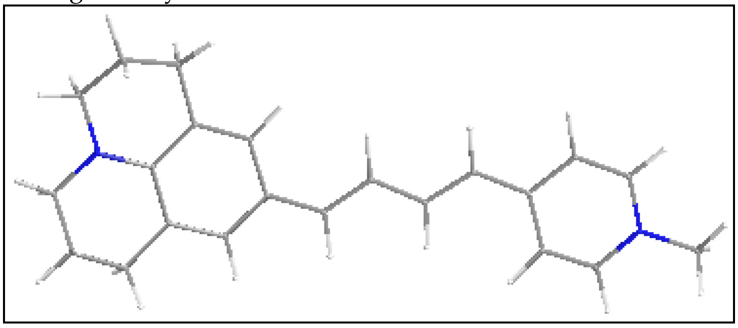

1 1

C	-1.30129500	-0.39572400	-0.06632700
C	-2.20086800	-1.49268000	-0.07024900
C	-3.57228700	-1.34091000	-0.08641900
C	-4.12887200	-0.01986700	-0.08184400
C	-3.23942500	1.10962500	-0.09788200
C	-1.87697400	0.90203300	-0.08097400
C	-4.48442600	-2.54989700	-0.11842000
C	-6.41359200	-0.96704800	-0.15073800
C	-5.84518700	-2.22461500	0.49893600
C	-6.08306100	1.50001000	-0.15460000
C	-5.19781900	2.56727700	0.48020800
C	-3.80426800	2.51440500	-0.14703900
H	-3.12300400	3.20786600	0.35889600
H	-5.65636400	3.55096500	0.33227000
H	-4.62868000	-2.87602100	-1.15941000
H	-1.79166700	-2.50171800	-0.06169700
H	-1.23028600	1.77578800	-0.08247400
H	-6.54762700	-3.05291000	0.35657900
H	-6.27058200	1.73854200	-1.21428200
H	-7.34648800	-0.67031100	0.33906800
H	-3.86739700	2.84897800	-1.19350400
H	-5.13417000	2.39306900	1.56185400
H	-7.05695900	1.46318800	0.34392800
H	-6.65002100	-1.15658400	-1.21044600
H	-5.74262500	-2.06222800	1.57941500
H	-4.01072800	-3.38811800	0.40506300
N	-5.48374600	0.16437700	-0.04795300
C	0.10165500	-0.64423100	-0.04701400
H	0.38629300	-1.69777500	-0.03811100
C	1.13745200	0.27307400	-0.03824800
H	0.92312700	1.34001200	-0.04607600
C	2.48913300	-0.12236400	-0.01943600
H	2.67688900	-1.19630700	-0.01197400
C	3.56548400	0.75027400	-0.01013200
H	3.35048300	1.81762600	-0.01687800
C	4.93698300	0.38956100	0.00766000
C	5.94699400	1.40270200	0.01798900

C	5.43413000	-0.95311200	0.01975300
C	7.27868300	1.08853600	0.03764600
H	5.66296600	2.45010700	0.01378900
C	6.77753200	-1.21204800	0.03941800
H	4.75849900	-1.80029900	0.01720900
N	7.70810100	-0.20981100	0.05306600
H	8.05417200	1.84608100	0.04455200
H	7.17048100	-2.22246100	0.04772000
C	9.14495400	-0.52274000	0.00262400
H	9.70367400	0.26159500	0.51628100
H	9.32604100	-1.47240200	0.50908300
H	9.48392100	-0.59220700	-1.03611500

TDDFT exited state energy 2.284 eV (542.83 nm)

4.1.2. Exited state geometry

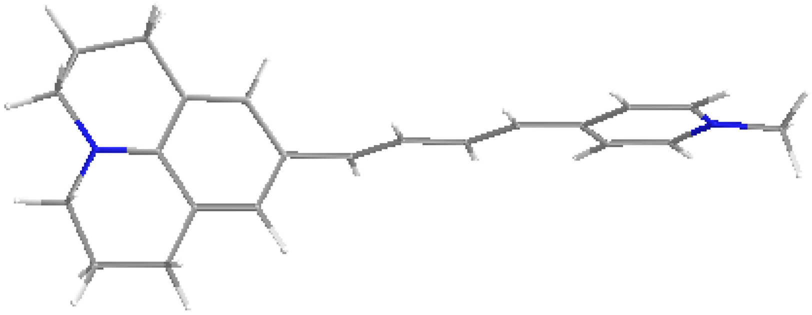

1 1

C	1.35632900	-0.01480500	-0.60512600
C	2.06701400	-1.21605400	-0.43295700
C	3.41575200	-1.24484800	-0.11258800
C	4.10905600	0.00515400	0.04240500
C	3.40168900	1.24501600	-0.12902100
C	2.05369800	1.19645400	-0.45014500
C	4.14040900	-2.55292600	0.08794800
C	6.18495900	-1.22920100	0.61894800
C	5.63642000	-2.40181800	-0.18453200
C	6.16680700	1.27084900	0.61318700
C	5.60964000	2.42773100	-0.20710600
C	4.11024200	2.56446000	0.05447400
H	3.66314400	3.31730100	-0.60202600
H	6.14203600	3.34336700	0.06738600
H	3.98873100	-2.89211700	1.12443800
H	1.53727600	-2.15616200	-0.55616900
H	1.51361800	2.12875800	-0.58688500
H	6.18140800	-3.30770900	0.09753600
H	6.09454200	1.48674900	1.69036700
H	7.23442800	-1.03777200	0.38228500
H	3.94827800	2.91838300	1.08441000
H	5.79791200	2.24628800	-1.27241400
H	7.22057300	1.09074200	0.38746900
H	6.12495700	-1.43519800	1.69883600
H	5.81596100	-2.23091600	-1.25312600
H	3.69888600	-3.32157900	-0.55378700
N	5.44358400	0.01491500	0.35519600
C	-0.09655200	-0.02470800	-0.97640100
H	-0.30275900	-0.03232400	-2.04599200
C	-1.10338100	-0.02242000	-0.08749000
H	-0.86226800	-0.01372700	0.97765000
C	-2.52282500	-0.02907800	-0.41149800
H	-2.78193300	-0.04152000	-1.46877800
C	-3.50011800	-0.01692300	0.54152300
H	-3.17987100	-0.00178900	1.58455800
C	-4.92282700	-0.02010700	0.34697300
C	-5.81468300	0.00733600	1.46112000
C	-5.56010200	-0.04774200	-0.93943900

C	-7.17253700	0.00766800	1.30359900
H	-5.41750300	0.02722000	2.47172500
C	-6.91876300	-0.04714100	-1.06094500
H	-4.97196200	-0.07471600	-1.84968200
N	-7.74970800	-0.03433300	0.04330500
H	-7.86211300	0.02932500	2.13840800
H	-7.41742700	-0.06687500	-2.02289100
C	-9.19376200	0.10398100	-0.11117900
H	-9.49377800	1.15986500	-0.12105700
H	-9.50755000	-0.36365400	-1.04724700
H	-9.70052100	-0.40128900	0.71497000

TDDFT exited state energy 1.6211 eV (764.80 nm)
